# NFAM1 Promotes Pro-Inflammatory Cytokine Production in Mouse and Human Monocytes

**DOI:** 10.3389/fimmu.2021.773445

**Published:** 2022-01-13

**Authors:** Kathryn W. Juchem, Anshu P. Gounder, Jian Ping Gao, Elise Seccareccia, Narayana Yeddula, Nicholas J. Huffmaster, Alexandra Côté-Martin, Steven E. Fogal, Donald Souza, Sarah Sirui Wang, Elizabeth R. A. Glynn, Ivy Yung, Julie Ritchie, Li Li, Jie Zheng, M. Lamine Mbow, Jun Li, Sumit K. Chanda

**Affiliations:** ^1^ Department of Immunology and Respiratory Disease Research, Boehringer Ingelheim Pharmaceuticals, Inc., Ridgefield, CT, United States; ^2^ Immunity and Pathogenesis Program, Infectious and Inflammatory Disease Center, Sanford Burnham Prebys Medical Discovery Institute, La Jolla, CA, United States; ^3^ Department of Biotherapeutics Discovery, Boehringer Ingelheim Pharmaceuticals, Inc., Ridgefield, CT, United States; ^4^ Department of Global Computational Biology and Digital Sciences, Boehringer Ingelheim Pharmaceuticals, Inc., Ridgefield, CT, United States

**Keywords:** NFAM1, Crohn’s disease, inflammatory bowel disease, monocytes, inflammation, CD40L, B cells, knockout mice

## Abstract

NFAT activating protein with ITAM motif 1 (NFAM1) is an ITAM bearing-transmembrane receptor that has been reported to play a role in B cell signaling and development. We performed expression analysis of NFAM1 using publicly available gene expression data sets and found that NFAM1 expression is significantly induced in intestinal biopsies from Crohn’s disease (CD) and ulcerative colitis (UC) patients. At the cellular level, we further observed high expression of NFAM1 in monocytes and neutrophils, and low expression in B and T cells. To explore the role of NFAM1 in multiple immune cells and its potential role in IBD, we generated NFAM1^-/-^ mice. In contrast with previous reports using NFAM1-transgenic mice, NFAM1^-/-^ mice have no obvious defects in immune cell development, or B cell responses. Interestingly, NFAM1^-/-^ monocytes produce reduced levels of TNF-α in response to activation by multiple IBD-relevant stimuli, including CD40L, TLR ligands and MDP. Additional cytokines and chemokines such as IL-6, IL-12, CCL3 and CCL4 are also reduced in CD40L stimulated NFAM1^-/-^ monocytes. Collectively, these findings indicate that NFAM1 promotes monocyte activation, thereby amplifying the response to diverse stimuli. Similarly, we observed that deletion of NFAM1 in human monocytes reduces expression of CD40L-induced CCL4. Lastly, to assess the role of NFAM1 in IBD, we compared development of anti-CD40 induced colitis in NFAM1^+/+^ and NFAM1^-/-^ mice. We found that although NFAM1 deletion had no impact on development of gut pathology, we did observe a decrease in serum TNF-α, confirming that NFAM1 promotes pro-inflammatory cytokine production *in vivo*. Taken together, we conclude that NFAM1 functions to amplify cytokine production and should be further evaluated as a therapeutic target for treatment of autoimmune disease.

## Introduction

NFAM1 is a transmembrane receptor that is expressed by cells of the innate and adaptive immune system. NFAM1 was discovered almost two decades ago *via* an *in silico* screen designed to find novel transmembrane receptors that contain both an extracellular immunoglobulin domain and a cytoplasmic ITAM (1). NFAM1 has been shown to activate nuclear factor of activated T-cells (NFAT), resulting in expression of TNF-α, IL-13 and IL-2 (1, 2). Calcineurin inhibitors, such as cyclosporine A and tacrolimus, block NFAT activation and have long been used to prevent transplant rejection. However, it is recently appreciated that NFAT inhibitors also have the potential to limit autoimmune disease [reviewed in Bendickova et al. (3)], as NFAT plays a prominent role in regulation of both innate and adaptive immunity. Unfortunately, NFAT inhibition also comes with the risk of increased susceptibility to infection. Inhibition of specific NFAT activating receptors such as NFAM1, therefore has the potential to limit immune pathology without inducing broad immunosuppression.

Increased expression of NFAM1 has recently been observed in multiple immune-related diseases, further suggesting that inhibition of NFAM1 may be of therapeutic value. For example, NFAM1 expression is upregulated in Paget’s disease of bone (PDB), a disease that is characterized by excessive bone resorption *via* abnormal osteoclasts (4). PDB may be triggered by viral infection, as is suggested by the detection of measles virus nucleocapsid protein (MVNP) in pagetic osteoclasts. *In vitro*, transfection of mouse bone marrow cells with MVNP results in a significant increase in NFAM1 expression, and NFAM1 shRNA reduces MVNP-induced osteoclast differentiation and bone resorption, suggesting that NFAM1 may play a role in PDB pathogenesis (4).

In further evidence of a link to disease, NFAM1 expression was recently reported to be upregulated in peripheral blood monocytes from patients with coronary artery disease (CAD) (5). Analysis of CAD monocytes revealed a strong correlation between NFAM1 and CCR2 expression. Notably, in CAD, CCR2 is known to promote recruitment of pathogenic monocytes to inflamed endothelium, exacerbating plaque formation and destabilization. In loss-of-function experiments, NFAM1 knockdown in monocytic cell lines correlated with decreased CCR2 expression and a corresponding decrease in MCP-1-mediated transendothelial migration. These findings suggest that NFAM1 may promote development of migratory, pathogenic monocytes and could serve as a biomarker for CAD (5).

Upon analysis of transcriptional data from IBD biopsies, we observed enhanced expression of NFAM1. We therefore sought to better understand NFAM1 function, particularly in the context of IBD. Knowledge of NFAM1’s *in vivo* function is currently limited to overexpression studies in which transfer of NFAM1 transgenic bone marrow progenitor cells into WT mice drastically impaired peripheral B cell development (2). However, to our knowledge, the impact of NFAM1 gene ablation in a whole animal model has not yet been evaluated. We therefore generated NFAM1^-/-^ mice and observed that, in contrast to results in overexpression studies, deletion of NFAM1 does not impair B cell development or maturation. However, NFAM1 deletion does impact monocyte function, as we observed that in comparison to NFAM1^+/+^ monocytes, NFAM1^-/-^ monocytes produce less TNF-α upon activation with IBD-relevant stimuli, such as CD40L. Correspondingly, we observed that deletion of NFAM1 in human monocytes also decreases CD40L-mediated cytokine production, demonstrating that our results are not limited to mouse monocytes. Lastly, in an anti-CD40 induced mouse model of IBD, NFAM1^-/-^ mice have decreased levels of serum TNF-α, further confirming that NFAM1 promotes inflammation.

## Methods

### Analysis of NFAM1 Expression in IBD Patient Datasets

Gene expression data from intestinal biopsies isolated from healthy controls and IBD patients were obtained from the Gene Expression Omnibus (GEO) web portal. GSE62207 dataset included the following samples: colonic Crohn’s disease (*n* = 51), ileal Crohn’s disease (*n*=208) and non-IBD controls (*n*=51) (6). GSE117993 dataset included the following samples: colonic Crohn’s disease (*n*=32), ileal Crohn’s disease (*n*=60), non-IBD control (*n*=55) and UC (*n*=43) (7). Analysis was done by ANOVA.

### Isolation of Human Monocytes for CRISPR-RNP Experiments

Human monocytes for CRISPR-RNP experiments were isolated from human peripheral blood that was obtained from San Diego Blood Bank. Protocols for collection and handling were approved by the Institution Review Board for human samples. Using the standardized Ficoll-Hypaque (GE Healthcare) density gradient centrifugation method, human peripheral blood mononuclear cells (PBMCs) were separated from whole blood and CD14 positive monocytes were isolated using CD14 binding beads by Miltenyi Biotech (following manufacturer’s protocols).

### Isolation of Human Immune Cell Subsets for Determination of NFAM1 Expression

T cells, B cells, NK cells and monocytes for NFAM1 expression experiments were isolated from human peripheral blood that was obtained from the Boehringer Ingelheim blood donor program. Using the standardized Ficoll-Hypaque (GE Healthcare) density gradient centrifugation method, PBMCs were isolated from whole blood. PBMCs were then frozen at -80 degrees Celsius until further use. T cells, B cells, NK cells and monocytes were isolated from thawed PBMCs *via* the use of Stemcell Technologies enrichment kits (#19051, #19054, #19055 and #19058, respectively). Neutrophils were isolated directly from human whole blood *via* the use of Stemcell Technologies neutrophil enrichment kit (#19257, RBC lysis method).

### Isolation of Mouse Immune Cell Subsets

Monocytes and neutrophils were isolated from bone marrow; T cells, B cells and NK cells were isolated from splenocytes according to the following protocols: femurs and tibias from rear legs were flushed with cell isolation media comprised of PBS (Gibco) supplemented with 2% heat inactivated fetal bovine serum and 1% Penicillin/streptomycin (both from Life Technologies). Bone marrow plugs were dispersed by repeated passage through a 27-gauge needle (BD) and filtered through a 40µm mesh strainer (Falcon). Monocytes and neutrophils were isolated *via* the use of Stemcell Technologies enrichment kits (#19861 and #19762, respectively). Spleens were disrupted using the blunt end of a syringe and filtered through a 40µm mesh strainer (Falcon). CD4 T cells, CD8 T cells, B cells and NK cells were isolated *via* the use of Stemcell Technologies enrichment kits (#18952, #19753, #19854, #19755, respectively).

### RNA Isolation

RNA was isolated *via* the use of RNeasy Plus Mini kit (Qiagen).

### RT-qPCR

RNA was converted to cDNA using EcoDry premix (Clonetech) according to the following protocol: 42 degrees Celsius for 60 minutes, 70 degrees Celsius for ten minutes and 4 degrees Celsius for 10 minutes. RT-qPCR was performed using Taqman universal PCR mix (Thermo Fisher) and the following Taqman probes: human GAPDH (Hs 02758991-g), human ACT-beta (Hs 99999903-m1), human HPRT1 (4332657), human NFMA1 (Hs00377608-m1), human NFAM1 (Hs 01066293-s1), mouse GAPDH (Mm99999915-g1), mouse HPRT1 (Mm03024075-m1) and mouse NFAM1 (Mm04336546-m1) (all from Thermo Fisher).

### Flow Cytometry

Splenocytes or stimulated monocytes were transferred to a 96 well polypropylene plate (Corning). Cells were incubated with LIVE/DEAD Fixable Aqua Dead Cell Stain (Molecular Probes) diluted to 0.1% in PBS for 15 minutes in the dark, washed twice with PBS and blocked for thirty minutes with TruStain FcX anti-mouse antibody (BioLegend) diluted to 0.1% in Cell Staining Buffer (BioLegend). For quantification of monocytes, dendritic cells and NK cells, splenocytes were stained with a cocktail of anti-CD4 clone RM4-5 in PerCP-Cy5.5, anti-CD8α clone 53-6.7 in PerCP-Cy5.5, anti-CD19 Clone 1D3 in PerCP-Cy5.5, anti-Ly6G clone 1A8 in PE, anti-CD11c clone HL3 in PE-Cy7, anti-NK1.1 clone PK136 in Alexa 700, anti-Ly6C clone AL-21 in APC-Cy7, and anti-CD11b clone M1/70 in eFlour 450. For quantification of CD4 and CD8 T cell subsets, splenocytes were stained with a cocktail of anti-CD4 clone GK1.5 in FITC, anti-CD25 clone PC61 in PE, anti-CD62L clone MEL-14 in PE-Cy7 and anti-CD8 clone 53-6.7 in Pacific Blue. For quantification of B cells, splenocytes were stained with a cocktail of anti-IgM clone RMM-1 in FITC, anti-B220 clone RA3-6B2 in PE, and anti-IgD clone 1A6-2 in APC. For characterization of monocytes post stimulation, cells were stained with the following FITC antibodies: anti-CD80 clone 16-10A1, anti-CD86 clone GL-1, anti-MHC II clone M5/114.15.2, anti-MHC I clone AF6-88.5, or anti-CD40 clone HM40-3 (BD). All antibodies were from BioLegend unless otherwise noted. Post staining, cells were washed twice with cell staining buffer, resuspended in 200 µL FluoroFix Buffer (BioLegend) and run on an LSRII Flow Cytometer (BD). Results were analyzed using FlowJo software.

### Stimulation of Mouse Monocytes

Monocytes from NFAM1^+/+^ and NFAM1^-/-^ mice (isolated as described above) were resuspended in cell culture media comprised of RPMI, 10% fetal bovine serum and 1% Penicillin-Streptomycin (all from Life Technologies). Monocytes were cultured in a 96 well tissue culture plate (Corning) at a concentration of 20,000 cells per well and either left untreated or pretreated for one hour with 100 µg/mL of mouse IFN-γ (R&D Systems) and subsequently stimulated with LPS (Sigma), MegaCD40L (Enzo), HKEB, HKLM, HKST, Pam3CSK4, Zymosan, FSL-1, or MDP (all from Invivogen). 24-48 hours post stimulation, supernatant was collected, and cells were harvested by flushing wells with cold PBS. Cells were analyzed by flow cytometry (see details above) and supernatants were analyzed by MSD for the presence of TNF-α (catalog #K152BHB-4), IL-6, IL-12, MIP-1α and MIP-1β (MSD custom U-plex).

### RNAseq

NFAM1^+/+^ and NFAM1^-/-^ monocytes were left unstimulated, stimulated with 50 ng/mL of IFN-γ alone or IFN-γ plus 1 µg/mL MegaCD40L. 6 or 24 hours later, supernatant was removed, cells were resuspended in RNAlater (Qiagen), and RNA was isolated as described above. Library construction and sequencing were performed at Beijing Genomics Institute as follows: RNA was fragmented, and cDNA was synthesized using the Illumina Truseq polyA mRNA library preparation protocol (non-stranded). RNA-sequencing was performed on an Illumina HiSeq2000 sequencer to generate 101x2 pair-end reads. RNAseq data were firstly QCed using fastQC, then aligned to the mouse reference genome using STAR algorithm. Rsubread was applied to generate raw counts, followed by data normalization by EdgeR. Differentially expressed genes (DEGs) and biological pathway were determined by LIMMA-Voom at fold change >=2, adjPvalue<0.05, and clusterProfiler at BioConductor, respectively.

### Human NFAM1 DNA Constructs

NFAM1 cDNA, NFAM1 cDNA with an N-terminal FLAG tag, NFAM1 cDNA with a C-terminal FLAG tag, chimeric construct composed of the extracellular and transmembrane domain of human CD8α fused to the cytoplasmic domain of either wildtype NFAM1 or NFAM1 in which the tyrosines of the ITAM were converted to phenylalanines were all cloned into pcDNA3.1 vectors. See **Supplemental Methods** for NFAM1 sequences.

### Transfection of 293 Cells With FLAG-Tagged NFAM1

293 cells (Invivogen) were cultured in Eagle’s Minimum Essential Medium, (ATCC) with 10% heat inactivated FBS (Gibco) and 1% Penicillin/streptomycin (Gibco). Cells were transfected with NFAM1 cDNA bearing either an N-terminal or C-terminal Flag tag *via* the use of TransIT-293 transfection reagent (Mirus) diluted in Opti-MEM media (Gibco). 48 hours post transfection, cells were harvested using 0.05% Trysin-EDTA (Invitrogen). Cells were stained for 15 minutes with 0.1% Zombie NIR dye (BioLegend) diluted in PBS and washed twice. For detection of cell surface expression, cells were immediately stained with anti-FLAG clone L5 in PE (BioLegend), washed twice, and resuspended in fixation buffer (BioLegend). For detection of intracellular expression, cells were incubated in stabilizing fixative (BD) for 20 min at RT, washed twice and resuspended in permeabilization buffer (BioLegend) before being stained with anti-FLAG clone L5 in PE (BioLegend). Flow cytometry and analysis was performed as described above.

### Nucleofection of Jurkat Cells With CD8α/NFAM1 Chimeric Constructs

Jurkat-Lucia™ NFAT cells (InvivoGen) were cultured in RPMI 1640 with 10 mM Hepes, 1 mM sodium pyruvate, 2 mM L-glutamine, 50 µg/mL Pen/Step, 10% heat inactivated fetal bovine serum (all from Life Technologies) plus 100 µg/mL Normocin (Invivogen). SE Cell Line 4D-Nucleofector^®^ X Kit L (Cat# V4XC-1024 from Lonza) was used according to manufacturer’s protocol to nucleofect Jurkat cells with vector alone, or CD8α/NFAM1 fusion constructs. Cells were rested for 24 hours, washed and moved either to a clean 96 well plate or to a plate that had been coated with 5 µg/mL of anti-CD8α antibody (Cat# BE0004-2, BioXCell). 24 hours later, supernatants were collected and analyzed using QUANTI-Luc Gold assay solution (InvivoGen) according to manufacturer’s protocol. Luminescence was measured on a TECAN Microplate Reader with a 0.1 second reading time.

### Generation of Thioglycolate-Induced Peritoneal Macrophages

3% Brewer’s thioglycolate (BD) was prepared in distilled water, autoclaved, and allowed to mature at least 2 weeks prior to use. Mice were given an intraperitoneal injection of 2 mL of 3% thioglycolate on day 0. On day 3, mice were sacrificed, and peritoneal lavage was performed using an 18 gauge needle and 10 mL of PBS with 1% FBS, 1% gentamycin (all from Gibco) and 1mM EDTA (Sigma). Cells were washed twice with PBS before being stimulated as described above (see mouse monocyte stimulation protocol).

### T Cell Recall Responses

OVA (Sigma) emulsified in complete Freund’s adjuvant (Sigma) was prepared as follows: OVA was diluted in saline to a concentration of 6 mg/mL and then emulsified 1:1 in CFA for a final OVA concentration of 3 mg/mL. Mice were anesthetized to surgical plane and were given 50 µL injections on either side of the base of the tail for a total volume of 100 µL and a total OVA dose of 0.3 mg. Ten days post immunization, animals were sacrificed and inguinal lymph nodes were collected. Lymph nodes were gently disrupted over a 100 µM cell strainer. Cells were washed twice with PBS and then cultured in RPM1 1640 with 10% FBS, 1x Penicillin-Streptomycin (Gibco) and 1x 2-mercaptoethanol (Sigma) and stimulated with 0, 11, 33.3 or 100 µg/mL of OVA. 3 days later, supernatant was collected and analyzed for production of IFN-γ, IL-17a and TNF-α *via* MSD mouse 4-plex (custom U-PLEX ELISA kit, catalog #K15069L).

### T Cell-Dependent and T Cell-Independent B Cell Responses

NFAM1^+/+^ and NFAM1^-/-^ mice were immunized with an NP-CGG (T-dependent antigen) or NP-LPS (T-independent antigen) and a secondary boost was given 21 days later. Blood samples were collected on days 0 (prior to immunization), 7, 14, 21, and 28. Serum was separated and stored at -80 degrees Celsius until further analysis. IgG ELISAs were performed on NP(23)-BSA coated plates using SBA Clonotyping System-C57BL/6-HRP (Catalog #5300-05B; Southern Biotech) according to manufacturer’s recommendations. The SBA Clonotyping System-C57BL/6-HRP kit is specifically designed for the isotyping of C57BL/6 mouse monoclonal antibodies (mouse IgA, mouse IgG1, mouse IgG2b, mouse IgG2c, mouse IgG3, and mouse IgM).

### CRISPR Nucleofection of Human Monocytes

Monocytes isolated from human PBMCs were nucleofected with CRISPR ribonucleoprotein (RNP) complexes using an Amaxa 4D nucleofection system according to manufacturer’s instructions (Lonza). Detailed protocols for RNP production have been previously published (8). Briefly, lyophilized guide RNA (gRNA) and tracrRNA (Dharmacon) were suspended at a concentration of 160 µM in 10 mM Tris-HCL, 150 mM KCl, pH 7.4. 5 µL of 160 µM gRNA was mixed with 5 µL of 160 µM tracrRNA and incubated for 30 min at 37 degrees Celsius. The gRNA:tracrRNA complexes were then mixed gently with 10 µL of 40 µM Cas9 (UC-Berkeley Macrolab) to form CRISPR-Cas9 ribonucleoproteins (crRNPs). gRNA sequences used are as follows:

Non-targeting control gRNA: GGCTCGTTCTACGCACTGA
NFAM1 gRNA: GCTGCCCGGGACCCTGCGAC.

Nucleofected monocytes were recovered with RPMI-1640 (Gibco) culture media supplemented with 10% heat-inactivated FBS (Gibco), 50 U/mL penicillin and 50 μg/mL streptomycin. Knockout efficiency was assessed by western blot detection of NFAM1 protein. Monocytes were stimulated with MegaCD40L (Enzo). Supernatant was collected at 24 hours and analyzed by luminex using a Human 38 plex kit (Millipore, catalog # HCYTMAG-60K-PX38).

### Generation of NFAM1 Knockout Mice

NFAM1^-/-^ mice were generated by injecting Cas9 protein along with proximal gRNA (CTAATTGTTCGGCCGGACGC) and distal gRNA (AGGGCAGCTACTCTCCCGAG) into C57BL/6NTac zygotes, resulting in deletion of exons 2 and 3. NFAM1^-/-^ and NFAM1^+/+^ mice were generated for experiments by intercrossing NFAM1^+/-^ mice. NFAM1^+/-^ mouse colony was maintained by crossing NFAM1^+/-^ mice to C57BL/6 mice from Taconic. NFAM1^-/-^ RAG2^-/-^ and NFAM1^+/+^ RAG2^-/-^ mice were generated for anti-CD40 colitis experiments by intercrossing NFAM1^-/-^ mice to RAG2^-/-^ mice (Taconic). All breeding and genotyping was performed at Taconic.

### Anti-CD40 Colitis

For induction of anti-CD40 colitis, 6-8 week old male NFAM1^-/-^ RAG2^-/-^ and NFAM1^+/+^ RAG2^-/-^ mice. were administered a 200 uL intraperitoneal injection of either 1mg/mL anti-CD40 (BioXCell clone FGK4.5) in 0.9% USP Sodium Chloride (NDC 0409-4888-06) or 0.9% Sodium Chloride alone. Mouse body weight was measured daily. Mice that reached 20% weight loss from baseline were administered 1mL of subcutaneous saline. Mice that reached 25% weight loss from baseline were euthanized. On day 7, mice were sacrificed and colons were collected for histology. TNF-α in serum was measured by V-Plex Plus Mouse TNF-α kit (MSD). IL-1β, IL-6 and IL-12/p70 were measured by custom MSD U-plex. All mouse experiments were performed in accordance with Boehringer Ingelheim Institutional Animal Care and Use Committee (IACUC) regulations.

### Histopathology

Histopathology score is determined with emphasis on the percentage of lesion involvement. Pathology captured are: 1) mucosa epithelium change (goblets loss, gland loss, and epithelium proliferation), 2) mucosa erosions/ulcerations, and 3) mucosa inflammation. Reported is the score from four sections from each animal for each parameter. Total score is the mean of all 3 parameters. Score scale is as follows: 0=normal; 1=minimal large focal area or minimal diffuse; 2=mild, diffuse mild, or multifocal affection 11-25%; 3=Moderate, 26 to 50% of mucosa affected with minimal to mild focal or multifocal changes; 4 = Marked, 51 to 75% of mucosa affected with mild to moderate change; 5 = Severe, 76 to 100% of mucosa affected with moderate to marked change.

### Western Blot

Cells were lysed in RIPA buffer (Thermo Fisher Scientific) with Halt Protease Inhibitor Cocktail (Thermo Fisher Scientific) and Phosphatase Inhibitor Cocktail (Sigma). Protein content was determined via Bradford protein assay kit (Thermo Fisher Scientific). Samples were run on NuPAGE, 4-12%, Bis-Tris, Mini Protein Gel and then transferred to an iBlot transfer stack using an iBlot transfer apparatus (Invitrogen) according to the Invitrogen Western Blot Technical Guide. Detection was performed according to the Li-COR Odyssey Western Blot Protocol, using α-human NFAM1 primary antibody (Sigma, cat#HPA031812) and donkey α-rabbit secondary antibody in IRDYE 800CW (LI-COR) in combination with an Odyssey Infrared Imager (LI-COR).

### Statistics

Statistics were calculated by either two-tailed student’s T Test (for experiments with a single comparison) or by ordinary one-way ANOVA (for experiments with multiple comparisons). Error bars depict standard deviation. Statistical significance is depicted as follows: **** indicates a P value of <0.0001, *** indicates a P value of <0.001, ** indicates a P value of <0.01, and * indicates a P value of <0.05.

### Data Availability

RNAseq data has been made publicly available at GSE188390.

## Results

### NFAM1 Is a Cell Surface Receptor That Is Upregulated in IBD and Highly Expressed in Monocytes and Neutrophils

**Figure 1:**
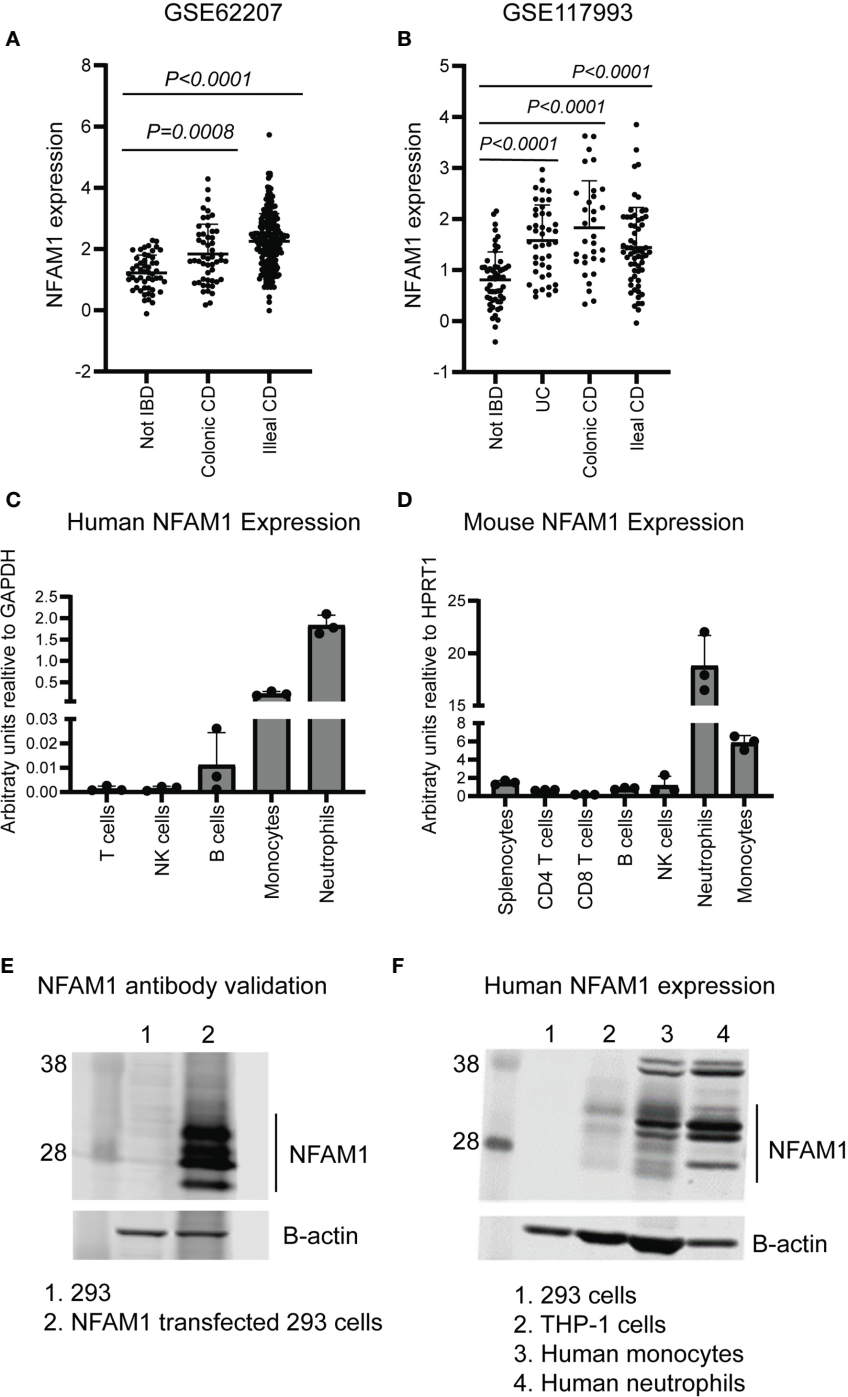
NFAM1 expression is increased in IBD and can be detected in cells of the innate and adaptive immune system. **(A, B)** NFAM1 mRNA expression in intestinal biopsies from non-IBD controls, colonic CD, ileal CD, or UC (data from publicly available datasets GSE62207 and GSE117993, see methods section for study description). **(C)** NFAM1 mRNA expression in human T cells, B cells, NK cells, monocytes and neutrophils. Shown is mean expression from three donors. Data is representative of two independent experiments. **(D)** NFAM1 mRNA expression in mouse splenocytes, CD4 T cells, CD8 T cells, B cells, NK cells, monocytes and neutrophils. Shown is mean expression from three independent mice. Data is representative of two independent experiments. In the second experiment (data not shown), samples were pooled from 10 individual mice. **(E)** NFAM1 protein expression in 293 cells or 293 cells transfected with NFAM1 cDNA. Results are representative of greater than 5 independent experiments. **(F)** NFAM1 protein expression in 293 cells, THP-1 cells, human monocytes and human neutrophils. Data is representative of three independent experiments from three independent donors.

Recent studies have linked NFAM1 expression to multiple diseases, including coronary artery disease and Paget’s disease of bone (4, 5). To explore the correlation between NFAM1 expression and IBD, we used publicly available datasets (6, 7) to assess NFAM1 mRNA expression in intestinal biopsies from pediatric IBD patients. We found that NFAM1 expression is increased in patients with ulcerative colitis (UC), colonic Crohn’s disease (CD), and ileal Crohn’s disease **(** **Figures 1A, B** **).** To identify the cell types that express NFAM1, we measured NFAM1 mRNA in human immune cells. As previously reported (1), NFAM1 mRNA is highly expressed in human neutrophils and monocytes, with low but detectable expression in T cells, NK cells and B cells **(** **Figure 1C** **).** Analysis of immune cells isolated from mouse spleen and bone marrow revealed a similar expression pattern **(** **Figure 1D** **)**.

We next sought to confirm the presence of NFAM1 protein. Prior to doing so, we validated a commercially available anti-NFAM1 antibody using 293 cells transfected with NFAM1 cDNA. Compared to non-transfected controls, we observed multiple bands of around 28 kDa, which match with NFAM1’s predicted molecular mass of 30 kDa **(** **Figure 1E** **).** The multiple bands are likely due to varying levels of glycosylation, as NFAM1 has a predicted N-glycosylation site within its extracellular immunoglobulin domain (1). Upon western blot analysis of primary human cells, we observed robust NFAM1 expression in neutrophils and monocytes, as was predicted based on mRNA expression. We also observed NFAM1 protein in the human monocytic THP-1 cell line **(** **Figure 1F** **).**

NFAM1 is reported to be an NFAT-activating Type I transmembrane receptor. (1) To verify cell surface expression of NFAM1, we transfected 293 cells with a full length NFAM1 construct bearing either an N-terminal or C-terminal FLAG tag. We detected cell surface expression of FLAG on cells that were transfected with the N-terminal but not the C-terminal tagged construct, while we detected intracellular FLAG in cells transfected with both constructs **(** **Supplemental Figures 1A, B** **).** As a control, we used western blot to verify that both constructs result in production of NFAM1 protein **(** **Supplemental Figure 1C** **)**. Next, we sought to confirm reports that NFAM1 activates NFAT. To do so, we employed an approach like that described by Ohtsuka et al, in which crosslinking of a chimeric protein containing the cytoplasmic domain of mouse NFAM1 resulted in activation of NFAT. In our experiments, we transfected NFAT reporter expressing Jurkat cells with chimeric cDNA constructs composed of the extracellular and transmembrane domains of human CD8α fused to the intracellular domain of either wildtype NFAM1 or NFAM1 with a functionally inactive ITAM. We found that transfection with the CD8α/wildtype NFAM1 construct resulted in spontaneous NFAT activation, which was further exacerbated by crosslinking with anti-CD8α **(** **Supplemental Figure 1D** **)**. In contrast, we did not observe NFAT activation with the CD8α/mutant NFAM1 constructs. In summary, we confirmed previous reports that NFAM1 is an NFAT activating cell surface receptor with an N-terminal extracellular domain and a C-terminal cytoplasmic domain.

### NFAM1^-/-^ Mice Have No Obvious Defects in Immune Cell Development or B Cell Activation

**Figure 2:**
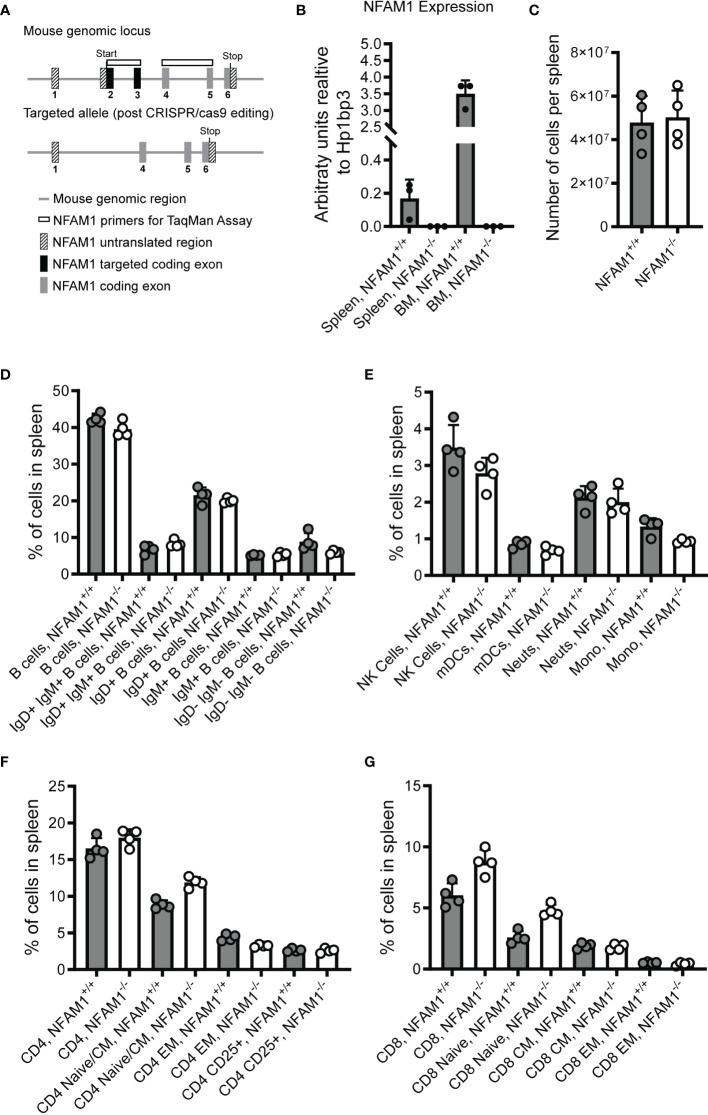
Analysis of spleens from NFAM1^+/+^ and NFAM1^-/-^ mice reveal no difference in percentages of innate and adaptive immune cells. Strategy for deletion of NFAM1 exons 2 and 3 *via* CRISPR-cas9 is shown in **(A)**. Mean expression of NFAM1 exons 2 and 3 in splenocytes and bone marrow from 3 NFAM1^+/+^ and 3 NFAM1^-/-^ mice is shown in **(B)**. Splenocytes were isolated from 4 NFAM1^+/+^ mice and 4 NFAM1^-/-^ mice. Mean number of cells per spleen in shown in **(C)**. Percentage of innate and adaptive immune cells were enumerated by flow cytometry. Mean percentage of B cells per spleen in shown in **(D)**, mean percentage of NK cells, myeloid dendritic cells (mDCs), neutrophils (neuts) and monocytes (mono) per spleen is shown in **(E)**, mean percentage of CD4 T cells is shown in **(F)** and mean percentage of CD8 T cells is shown in **(G)**. **(C–G)** Results are representative of two independent experiments. In the second experiment (data not shown), spleens from 9 NFAM1^-/-^ and 6 NFAM1^+/+^ mice were analyzed with no statistically significant differences in the percentage of innate or adaptive immune cells between NFAM1^+/+^ and NFAM1^-/-^ mice.

To date, only a limited number of papers have been published on NFAM1 (1, 2, 4, 5, 9), none of which have used knockout mice as a means of understanding NFAM1 function. To this end, we generated NFAM1^-/-^ mice by using CRISPR-cas9 to delete exons 2 and 3 **(** **Figure 2A** **).** These mice fail to produce the most common NFAM1 transcript (NCBI transcript NM_001271413), which utilizes a translation initiation codon contained within exon 2. However, there are reports of additional NFAM1 transcripts that utilize an upstream translation initiation codon within exon 1 (NM_028728_3, NM_001271411_1, NM_001271412_1, XM_006521486_2). For these transcripts, removal of exons 2 and 3 results in a partial loss of NFAM1’s extracellular Ig-like domain. In addition, removal of exons 2 and 3 introduces a frameshift from exon 1 to exon 4-6.

We used RT-qPCR to confirm deletion of exons 2 and 3 **(** **Figure 2B** **),** as we found that commercially available anti-human NFAM1 antibodies fail to cross-react with mouse NFAM1. Additionally, attempts to generate our own antibodies by immunizing rabbits with a mouse NFAM1 Fc fusion protein were unsuccessful (data not shown). After confirming deletion at the mRNA level, we performed flow cytometry to determine if NFAM1 deletion impacts immune cell development. It was previously reported that transfer of NFAM1 overexpressing bone marrow progenitor cells into WT mice results in drastically reduced numbers of mature splenic B cells (2). In contrast, we found that NFAM1^-/-^ mice had normal numbers of cells per spleen **(** **Figure 2C** **)** and no change in the percentage of IgM positive, IgD positive, IgM/IgD double positive or IgM/IgD double negative B cells **(** **Figure 2D** **)**. These findings indicate that although overexpression of NFAM1 may disrupt B cell maturation, endogenous NFAM1 does not play a significant role in B cell development. Similarly, we found that NFAM1^-/-^ mice had no significant changes in the percentage of splenic dendritic cells, monocytes, neutrophils, NK cells or CD4^+^ or CD8^+^ naïve, central memory, or effector memory T cells **(** **Figures 2E–G** **).**

**Figure 3:**
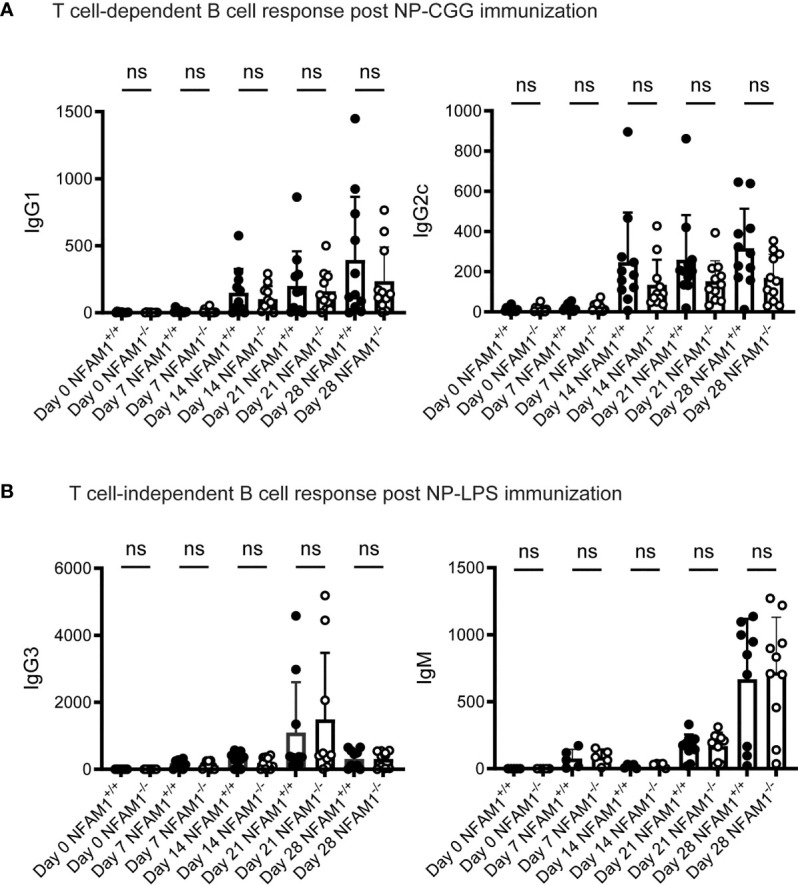
NFAM1^-/-^ mice do not have significant defects in T cell-dependent or T cell-independent B cell responses. **(A)** Mice were immunized with NP-CGG on days 0 and 21. Shown is serum IgG1 and IgG2c. Data was combined from two independent experiments for a total of 11 NFAM1^+/+^ and 11 NFAM1^-/-^ mice per timepoint. **(B)** Mice were immunized with NP and LPS on days 0 and 21. Shown is serum IgG3 and IgM. Data was combined from two independent experiments. Shown is mean of 6 NFAM1^+/+^ mice and 6 NFAM1^-/-^ mice on days 0, 7 and 14 and mean of 10 NFAM1^+/+^ and 10 NFAM1^-/-^ mice on days 21 and 28. NS indicates that the difference between NFAM1^+/+^ and NFAM1^-/-^ samples is not statistically significant.

Although we did not observe a role for NFAM1 in promoting B cell development, we were curious if NFAM1 promotes B cell-mediated antibody production. To determine if NFAM1^-/-^ mice mount normal T cell-independent and T cell-dependent B cell responses, we immunized mice on days 0 and 21 with the hapten (4-hydroxy-3-nitrophenyl) acetyl (NP) conjugated to LPS or chicken γ-globulin (CGG). Immune responses were assessed at multiple timepoints by measuring NP-specific antibody titers. All immunized mice exhibited similar levels of NP-specific IgG1 and IgG2c production following immunization with the T-dependent antigen, NP-CGG **(** **Figure 3A** **).** Similarly, NFAM1^+/+^ and NFAM1^-/-^ mice showed no statistically significant difference in NP-specific IgG3 and IgM production after stimulation with the T-independent antigen, NP-LPS **(** **Figure 3B** **).** These finding indicate that, in addition to not impacting B cell development, endogenous NFAM1 has little impact on B cell activation or antibody generation.

### NFAM1^-/-^ Monocytes Exhibit Reduced Pro-Inflammatory Cytokine Production in Response to Multiple IBD-Relevant Stimuli

**Figure 4:**
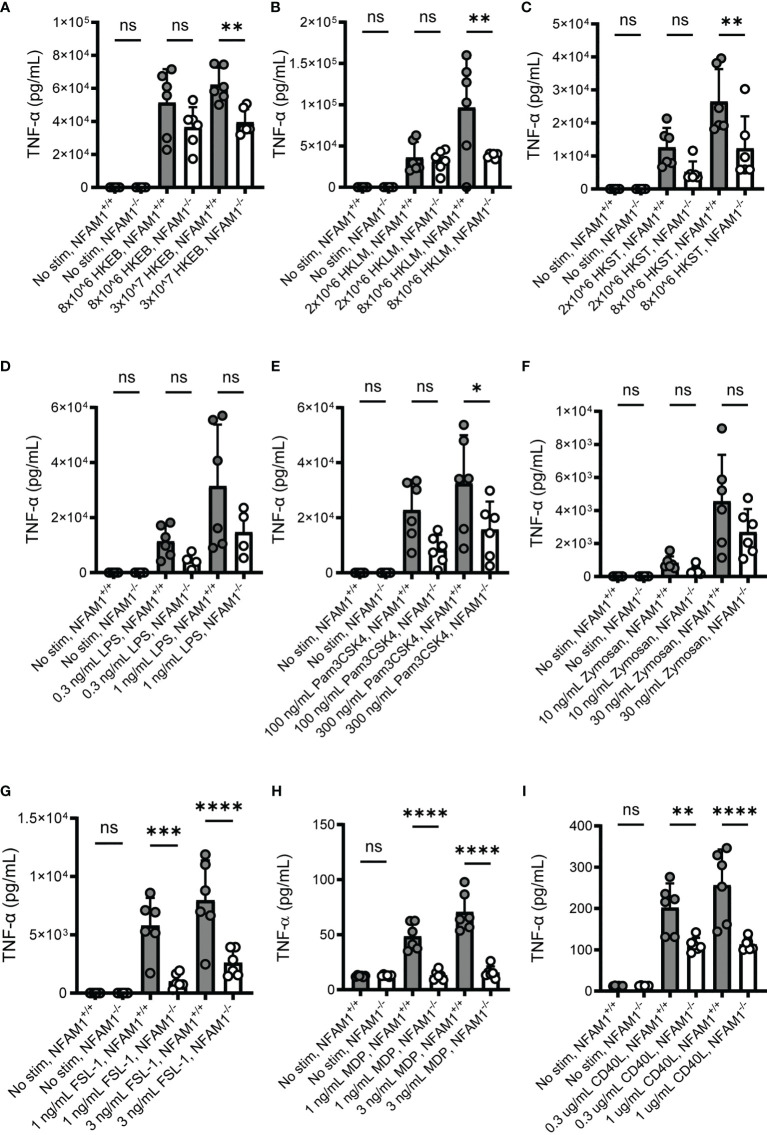
NFAM1^-/-^ monocytes produce reduced TNF-α in response to activation with multiple IBD-relevant stimuli. Monocytes were isolated from 6 NFAM1^+/+^ mice and 6 NFAM1^-/-^ mice. Monocytes were primed with IFN-γ and stimulated with HKEB, HKLM, HKST, LPS, Pam3CSK4, Zymosan, FSL-1, MDP and CD40L for 48 hours. Mean TNF-α production is shown in **(A–I)**. HKEB, HKLM, HKST, Pam3CSK4 and FSL-1 data are representative of two independent experiments in which NFAM1^-/-^ monocytes produced reduced TNF-α in response to at least one concentration of each stimuli. LPS and Zymosan data are representative of 3 independent experiments in which there was consistently no statistically significant difference between TNF-α production in NFAM1^+/+^ and NFAM1^-/-^ monocytes. MDP and CD40L data are representative of 3 independent experiments in which NFAM1^-/-^ monocytes produced reduced TNF-α in response to multiple concentrations of MDP and CD40L. Statistical significance is depicted as follows: **** indicates a P value of <0.0001, *** indicates a P value of <0.001, ** indicates a P value of <0.01, * indicates a P value of <0.05 and ns indicates the comparison is not statistically significant.

We next investigated the role of NFAM1 in monocyte activation, as monocytes express high levels of NFAM1 and knockdown of NFAM1 in a monocytic cell line has been shown to impact functional readouts, such as chemotaxis (5). Because we are interested in the role of NFAM1 in IBD, we tested the impact of NFAM1 deletion on the response to microbial stimuli, as gut dysbiosis is a major driver of IBD pathogenesis (10). To do so, we isolated monocytes from the bone marrow of NFAM1^+/+^ and NFAM1^-/-^ mice, primed the monocytes with IFN-γ and then stimulated the cells with whole bacteria or microbial components. We found that NFAM1^-/-^ monocytes exhibited reduced production of TNF-α in response to stimulation with heat-killed *Escherichia coli* (HKEB), heat-killed *Listeria monocytogenes* (HKLM) and heat-killed *Salmonella typhimurium* (HKST) **(** **Figures 4A–C** **).** We observed a trend towards decreased TNF-α production with the TLR4 ligand LPS and the dectin-1 ligand depleted zymosan **(** **Figures 4D, F** **),** but these differences did not reach statistical significance. Importantly, we observed a marked decrease in TNF-α production in response to stimulation with the TLR1/2 ligand Pam3CSK4, the TLR2/6 ligand FSL1, and the NOD2 ligand MDP **(** **Figures 4E, G, H** **).** The decreased response to MDP holds particular significance for IBD, as mutations of *CARD15*/NOD2 have been linked to CD (11). Lastly, we investigated the response to CD40L, as *in vivo* administration of anti-CD40 has been shown to induce IBD-like pathology in mice (12) and CD40L is expressed by T cells which are known to play a significant role in IBD pathogenesis (13). As was observed with microbial stimuli, NFAM1^-/-^ monocytes also produced reduced TNF-α upon stimulation with CD40L (**Figure 4I** **).**

In summary, these data indicate that NFAM1 on mouse monocytes promotes TNF-α production in response to multiple IBD-relevant stimuli. However, given that monocytes were primed with IFN-γ, which is known to potentiate pro-inflammatory cytokine production (14), it is also possible that NFAM1 regulates IFN-γ responsiveness, with no direct effect on the response to microbial stimuli or CD40L. To further dissect the role of NFAM1, we stimulated monocytes with IFN-γ alone, CD40L alone, or IFN-γ plus CD40L. We found that IFN-γ potentiates CD40L-induced TNF-α production in both NFAM1^+/+^ and NFAM1^-/-^ monocytes. In contrast, we observed reduced TNF-α production in NFAM1^-/-^ monocytes stimulated with both CD40L alone and IFN-γ plus CD40L **(** **Supplemental Figure 2** **).** These results indicate that the decreased TNF-α that we observed in response to multiple stimuli is not due to defective IFN-γ priming.

**Figure 5:**
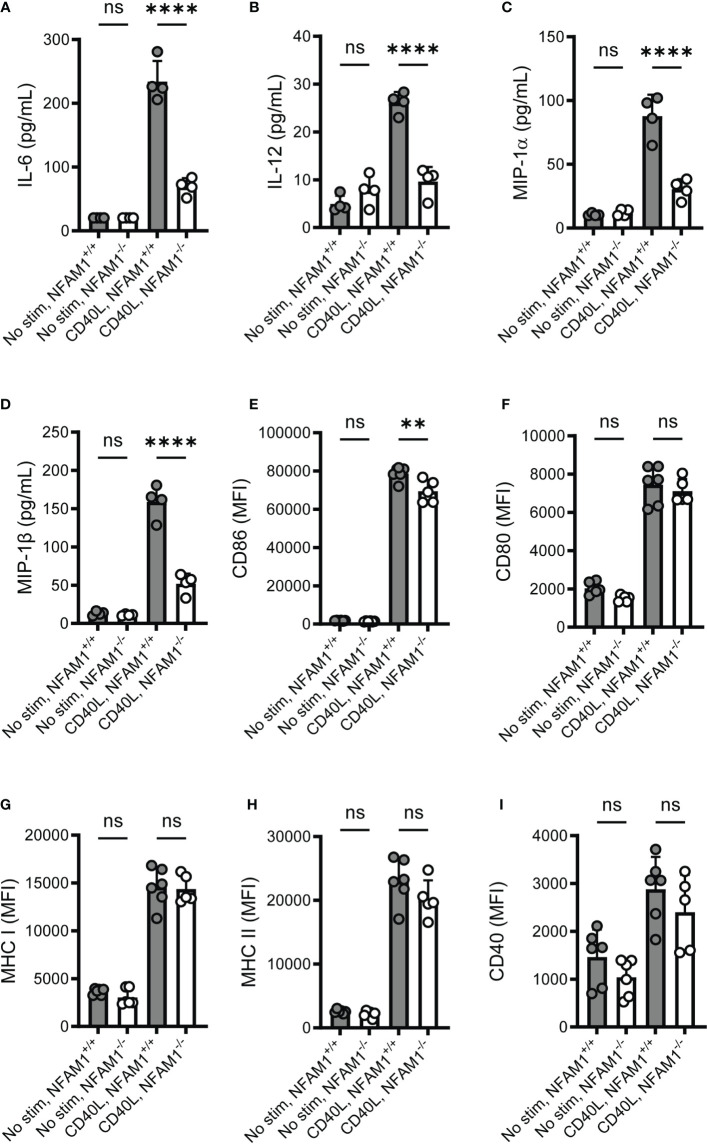
Upon stimulation with CD40L, NFAM1^-/-^ monocytes exhibit reduced production of multiple pro-inflammatory cytokines and chemokines but have little to no defect in upregulation of cell surface activation markers. **(A–D)** Monocytes from 4 NFAM1^+/+^ and 4 NFAM1^-/-^ mice were primed with IFN-γ and stimulated with CD40L for 48 hours. Mean levels of IL-6, IL-12, MIP-1α and MIP-1β is shown in **(A–D)**, respectively. **(E–I)**. Monocytes from 6 NFAM1^+/+^ and 6 NFAM1^-/-^ mice were primed with IFN-γ and stimulated with CD40L for 24 hours. Mean expression of CD86, CD80, MHC-I, MHC-II and CD40 is shown in **(E–I)** respectively. Cytokine and chemokine data are representative of three independent experiments. Cell surface expression data are representative of two independent experiments. Statistical significance is depicted as follows: **** indicates a P value of <0.0001, ** indicates a P value of <0.01, and ns indicates the comparison is not statistically significant.

We next sought to determine if NFAM1 impacts monocyte activation beyond production of TNF-α, such as production of additional cytokines and chemokines and upregulation of cell surface markers. We focused on the response to IFN-γ plus CD40L, as these stimuli result in a highly significant difference in TNF-α production between NFAM1^+/+^ and NFAM1^-/-^ monocytes. Upon stimulation with IFN-γ plus CD40L, we found that NFAM1^-/-^ monocytes produce significantly reduced IL-6, IL-12, MIP-1α/CCL3 and MIP-1β/CCL4 **(** **Figures 5A–D** **).** In contrast, upon analysis of cell surface marker expression, we observed only a minor decrease in upregulation of CD86 and no difference in upregulation of CD80, MHC-I, MHC-II and CD40 **(** **Figures 5E–I** **)**, indicating that NFAM1 modulates only some aspects of CD40L-induced monocyte activation.

**Figure 6:**
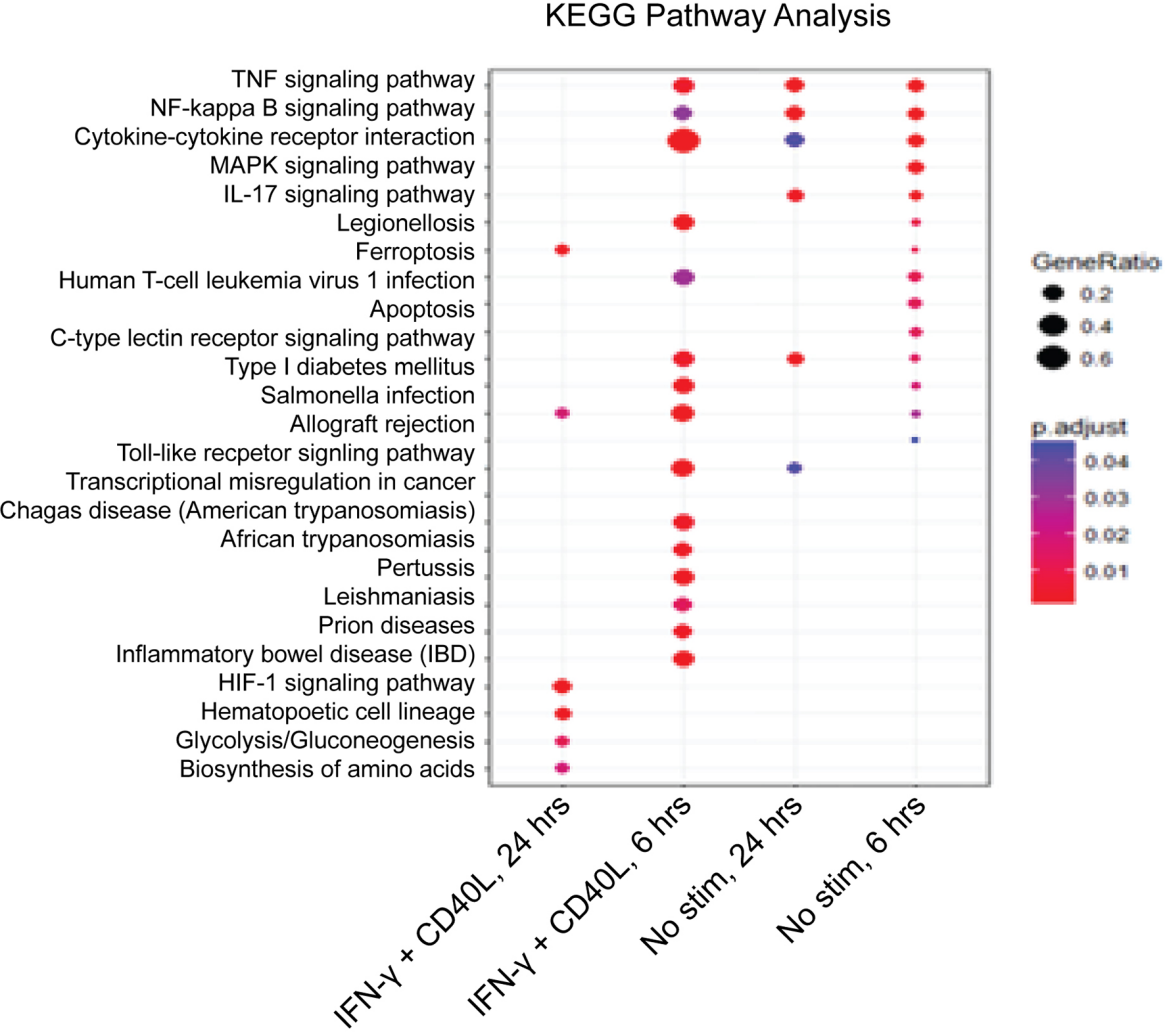
KEGG pathway analysis confirms differential gene expression between NFAM1^+/+^ and NFAM1^-/-^ monocytes. Monocytes from 4 NFAM1^+/+^ and 6 NFAM1^-/-^ mice were left unstimulated or stimulated with IFN-γ and CD40L. RNA was isolated and analyzed by RNAseq at 6 and 24 hours. Shown is KEGG analysis of genes with increased expression in NFAM1^+/+^ monocytes versus NFAM1^-/-^ monocytes.

**Figure 7:**
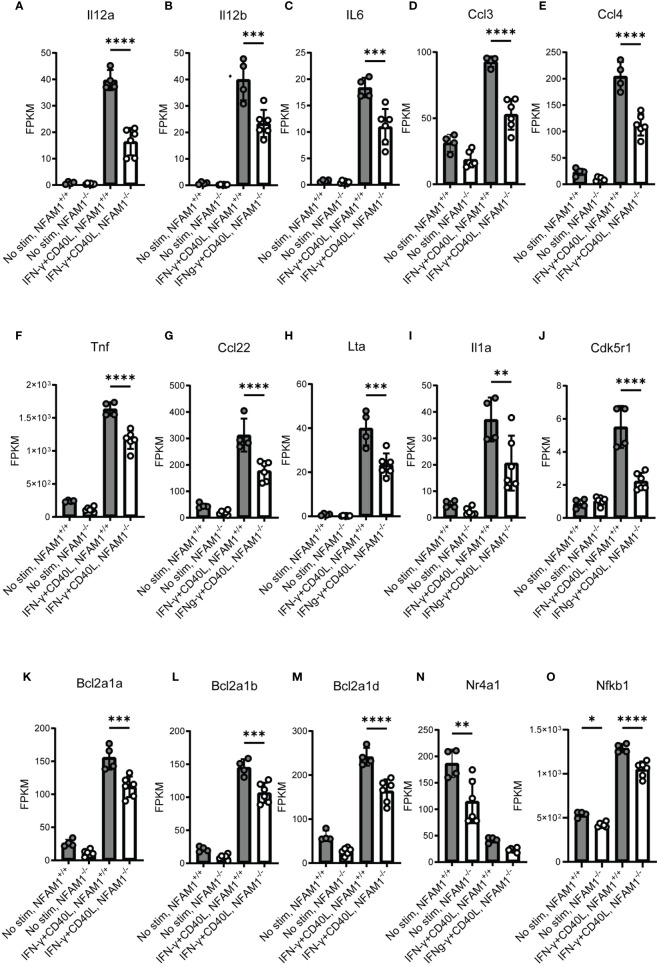
RNAseq analysis confirms that NFAM1^-/-^ monocytes have reduced expression of CD40L-induced pro-inflammatory cytokines and chemokines. RNAseq was performed as described in **Figure 6**. Mean FPKM for select genes at 6 hours is shown in **(A–O)**. Statistical significance is depicted as follows: **** indicates a P value of <0.0001, *** indicates a P value of <0.001, ** indicates a P value of <0.01, and * indicates a P value of <0.05.

To better understand the impact of NFAM1 deletion on monocyte activation, we performed whole transcriptome analysis on monocytes that were cultured in the presence or absence of IFN-γ plus CD40L for 6 or 24 hours. KEGG pathway enrichment analysis revealed that compared to NFAM1^+/+^ monocytes, NFAM1^-/-^ monocytes that are stimulated with IFN-γ plus CD40L for 6 hours have decreased expression of genes associated with multiple pathways, including IBD, TNF signaling, NF-kB signaling and cytokine-cytokine interaction **(** **Figure 6** **)**. Notably, at the 6-hour timepoint, RNAseq data confirmed what we had observed at the protein level, with NFAM1 deletion reducing CD40L-induced expression of Il-12, Il-6, Ccl4, Ccl3 and TNF **(** **Figures 7A–F** **)**, but having only a very minor impact on expression of CD86 and CD40 **(** **Supplemental Figures 3B, C** **)** and no impact on expression of CD80, H2-D1 and H2-Q7 **(** **Supplemental Figures 3A, D, E** **)**. Furthermore, we observed that NFAM1 promotes CD40L-induced expression of additional pro-inflammatory mediators, including Il-1a, Ccl22 and lymphotoxin-α (LTA), and Cdk5r1 **(** **Figures 7G–J** **).** Beyond regulation of pro-inflammatory mediators, NFAM1^-/-^ monocytes also had decreased CD40L-induced expression of the anti-apoptotic molecules Bcl2a1a, Bcl2a1b and Bcl2a1d **(** **Figures 7K–M** **).** Finally, unstimulated NFAM1^-/-^ monocytes had a small but significant decrease in expression of transcription factors including Nfkb1 and Nr4a1 **(** **Figures 7N, O** **).**

Having observed that NFAM1 promotes production of IL-12, we were curious to see if NFAM1^-/-^ mice had impaired ability to produce the Th1-associated cytokine IFN-γ, as IL-12 is known to drive T cell differentiation towards a Th1 phenotype. We therefore immunized NFAM1^+/+^ and NFAM1^-/-^ mice with OVA emulsified in CFA and performed *ex-vivo* restimulation on day 10. We did not observe decreased production of IFN-γ, IL-17 or TNF-α by cells isolated from NFAM1^-/-^ mice **(** **Supplemental Figure 5** **),** indicating that under these immunization conditions, NFAM1 does not promote either Th1 or Th17 cell development. Monocytes are only one of many cell types that can produce IL-12. It is therefore possible that the lack of defect in Th1 cell development is a result of compensatory IL-12 production by other myeloid cell types. To test if NFAM1 deletion impacts pro-inflammatory cytokine production in macrophages, we isolated thioglycolate-induced peritoneal macrophages from NFAM1^+/+^ and NFAM1^-/-^ mice and stimulated the cells with IFN-γ and CD40L. We found that peritoneal macrophages from NFAM1^-/-^ mice produced reduced levels of TNF-α **(** **Supplemental Figure 4** **),** although the magnitude of the decrease was not as dramatic as that observed in NFAM1^-/-^ monocytes.

**Figure 8:**
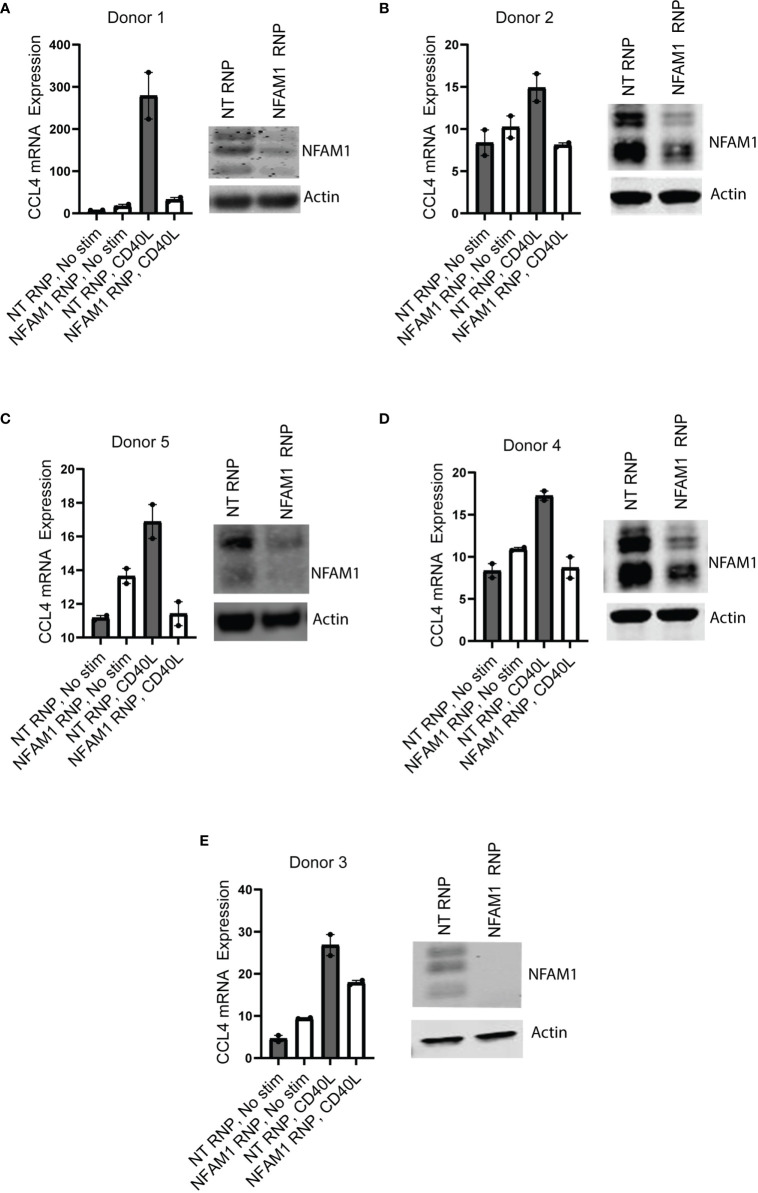
NFAM1 promotes CD40L-induced CCL4 production in human monocytes. **(A–E)** Human monocytes were isolated from 5 independent donors and treated with Non-targeting (NT) CRISPR-RNP or NFAM1 CRISPR-RNP. Cells were plated in duplicate and stimulated with CD40L. Shown are NFAM1 protein expression and CCL4 mRNA expression.

We next asked if NFAM1 promotes production of pro-inflammatory cytokines and chemokines in human monocytes. To do so, we used CRISPR-RNP to delete NFAM1, which resulted in significantly reduced NFAM1 protein expression as measured by western blot. Using mRNA expression as a readout, we observed that NFAM1 CRISPR-RNP reduced CD40L-mediated induction of CCL4 mRNA expression in five independent donors **(** **Figure 8** **).** Furthermore, in a 6^th^ donor, we demonstrated that NFAM1 CRISPR-RNP reduced CD40L-mediated production of MIP-1β/CCL4, TNF-α, IL-8, IL-1β, IL-1Ra and CCL22 protein **(** **Supplemental Figure 6** **).** These findings indicate that the role of NFAM1 in promoting monocyte activation is conserved between mouse and human. Furthermore, it suggests that the decreased ability of NFAM1^-/-^ monocytes to respond to stimuli such as CD40L is not the result of a developmental defect, as we were able to replicate these findings using acute deletion of NFAM1 in human monocytes.

### In Anti-CD40 Induced Colitis Model, NFAM1^-/-^ Mice Exhibit Decreased Levels of Serum TNF-α

**Figure 9:**
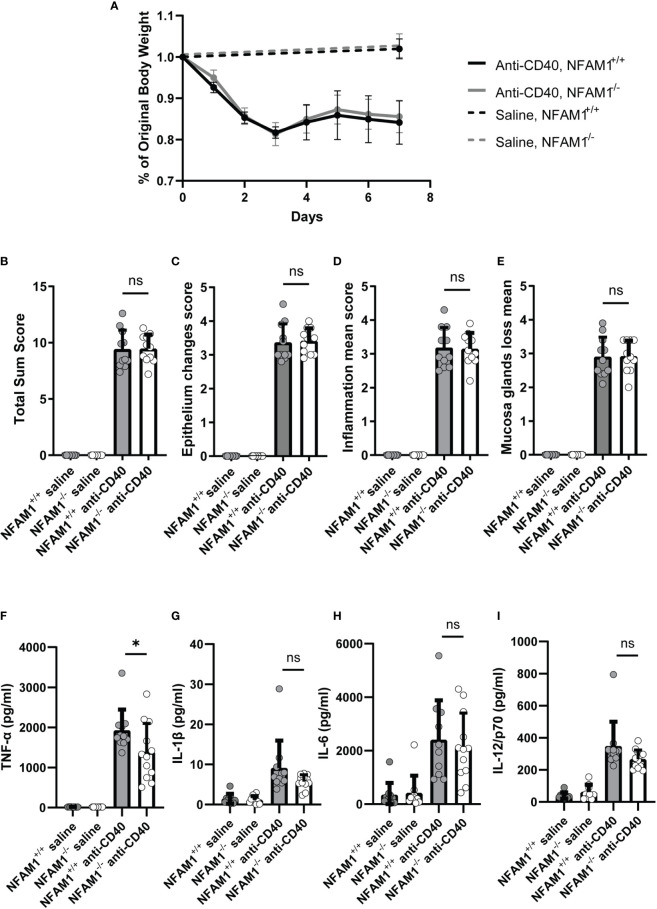
Induction of anti-CD40 colitis in NFAM1^-/-^ mice results in decreased production of TNF-α but no significant difference in development of gut pathology. **(A–I)** 9 NFAM1^+/+^ RAG2^-/-^ mice and 9 NFAM1^-/-^ RAG2^-/-^ mice were injected with saline and weighed on days 0 and 7. 11 NFAM1^+/+^ RAG2^-/-^ mice and 12 NFAM1^-/-^ RAG2^-/-^ mice were injected with anti-CD40 and weighed daily on days 0-7. Mean percent of original body weight is shown in **(A)**. Mean gut sum pathology scores are shown in **(B)**, mean epithelial change scores are shown in **(C)**, mean inflammation scores are shown in **(D)**, mean mucosal gland loss scores are shown in **(E)**. Serum TNF-α, IL-1β, IL-6 and IL-12 are shown in **(F–I)**, respectively. Data are representative of two independent experiments. In the second experiment (data not shown), there was also a statistically significant decrease in anti-CD40 induced TNF-α production in NFAM1^-/-^ mice. Statistical significance is depicted as follows: * indicates a P value of <0.05 and ns indicates the comparison is not statistically significant.

Lastly, we investigated the role of NFAM1 in promoting colitis *in vivo*. We chose the anti-CD40 model, which is commonly used to study the role of the innate immune system in promoting intestinal inflammation and is known to be driven by production of IL-1β, IL-23 and IL-12 by myeloid cells (12). This model is an obvious choice, given that NFAM1^-/-^ monocytes produce reduced IL-12 upon stimulation with CD40L. The anti-CD40 colitis model is run on a RAG^-/-^ background, we therefore first generated NFAM1^+/+^ RAG^-/-^ and NFAM1^-/-^ RAG^-/-^ mice. Upon administration of anti-CD40, NFAM1 does not appear to promote colon pathology as measured by weight loss, epithelial changes, mucosal gland loss and inflammation **(** **Figures 9A–E** **).** We also did not observe a statistically significant decrease in serum levels of IL-1β, IL-6, or IL-12 **(** **Figures 9G–I** **).** We did, however, observe a decrease in the level of serum TNF-α in NFAM1^-/-^ mice that were injected with anti-CD40 compared to NFAM1^+/+^ controls **(** **Figure 9F** **).** These findings indicate that although NFAM1 is not a major driver of intestinal damage in the anti-CD40 model, NFAM1 does have an impact on production of pro-inflammatory TNF-α.

## Discussion

The main goal of the present study was to provide insight into the role of NFAM1 in IBD, as we observed that NFAM1 expression is significantly induced in IBD patient biopsies. Our approach was to generate NFAM1^-/-^ mice as a means of better understanding NFAM1 function. We observed that NFAM1 promotes production of pro-inflammatory cytokines and chemokines in CD40L-stimulated mouse and human monocytes as well as mouse peritoneal macrophages. Despite the role of NFAM1 in promoting CD40L-mediated activation, NFAM1^-/-^ mice are not protected against development of anti-CD40 colitis. However, we did observe decreased levels of serum TNF-α, suggesting that although NFAM1 is not a major driver of IBD, NFAM1 does promote inflammation and therefore has the potential to contribute to human disease.

To our knowledge, the present study is the first to phenotype NFAM1^-/-^ mice. Notably, we observed that NFAM1^-/-^ mice have normal numbers of splenic B cells and no obvious defects in B cell-mediated antibody production. These observations are significant because NFAM1 is routinely referred to as a regulator of B cell signaling (4, 5, 15–17). The association of NFAM1 with B cell signaling came from a study by Ohtsuka et al. in which NFAM1 was reported to block B cell development (2). The difference in results from our study and those of Ohtsuka et al. are likely due to the distinct methodologies used to investigate NFAM1 function. While we investigated the impact of deleting NFAM1, Ohtsuka et al. generated transgenic mice that overexpress NFAM1 in the hematopoietic compartment. One important limitation of the latter approach is that overexpression of transmembrane receptors can induce spontaneous clustering that promotes ligand-independent signal transduction. Therefore, evidence that overexpression of NFAM1 blocks B cell development should not be interpreted as proof that endogenous NFAM1 plays a similar role. While we cannot definitively conclude that NFAM1 has no B cell intrinsic activity, as we performed a relatively crude analysis of peripheral B cell subsets as well as B cell-mediated antibody production, our results strongly suggest that NFAM1 does not play a major role in B cell function.

A second notable finding from the present studies is the observation that NFAM1 promotes monocyte-mediated inflammation. Studies by Long et al. also report that NFAM1 plays an activating role on monocytes (5). However, while we observed that NFAM1 promotes CD40L-induced production of cytokines and chemokines, Long et al. report that NFAM1 promotes expression of chemokine receptors, including CCR2, CCR5 and CX3CR1, none of which are decreased in NFAM1^-/-^ mouse monocytes. The difference in results of our studies and those of Long et al. could be due to differences in the cell types that were utilized. Experiments by Long et al. were performed in U-937 and THP-1 cell lines. In contrast, our studies utilized primary mouse and human monocytes and therefore may more accurately depict the role of NFAM1 in human disease. Additionally, Long et al. used shRNA and siRNA as a means of knocking down NFAM1, while we generated NFAM1^-/-^ mice. An advantage of using knockout mice is that they provide a homogenous population of monocytes with complete knockdown of NFAM1. Regardless of the differences in the specific target genes that were modulated by NFAM1, both our results and those of Long et al. indicate that NFAM1 promotes monocyte activation, warranting further investigation of the role of NFAM1 in human disease.

In the present studies, we provide evidence that NFAM1 deletion reduces pro-inflammatory cytokine production by CD40L-stimulated monocytes. We also observed that NFAM1 deletion causes a significant, although less profound, reduction in TNF-production by CD40L-stimulated peritoneal macrophages. In comparison to monocytes, the relatively subtle impact of NFAM1 deletion in peritoneal macrophages may be a result of the cells being generated in the highly pro-inflammatory environment that is induced by intraperitoneal injection of thioglycolate. It is possible that this pro-inflammatory environment over-rides the need for NFAM1. Additional experiments in bone marrow-derived macrophages will provide further insight into the role of NFAM1 in activation of macrophages and will be the subject of future studies.

In addition to monocytes and macrophages, NFAM1 is expressed by multiple cell types that were not the focus of this study. For instance, we observed that NFAM1 is highly expressed by both mouse and human neutrophils **(** **Figures 1C, D** **)** and NFAM1 is reported to be expressed by both osteoclasts and pre-osteoclasts. In a measles virus protein-mediated *in vitro* model of PDB, NFAM1 promotes osteoclast development and bone resorption (4). Interestingly, similar to our observations in NFAM1^-/-^ monocytes, NFAM1 shRNA reduces RANKL-induced expression of IL-6 and TNF-α in the preosteoclast cell line RAW264.7 (9). Together these findings suggest that NFAM1 promotes pro-inflammatory cytokine and chemokine expression in multiple cell types.

An important remaining question is how does NFAM1 promote production of pro-inflammatory cytokines and chemokines? NFAM1 is a transmembrane receptor that contains a cytoplasmic ITAM. Upon over-expression in cell lines, we and others (1, 5) have reported that NFAM1 triggers activation of the calcium-activated transcription factor NFAT. (18) In further support of a link between NFAM1 and NFAT, knockdown of NFAM1 in pre-osteoclasts reduces intracellular calcium oscillations and NFATc1 activation, thereby reducing osteoclast development (4, 9) Interestingly, CD40L stimulated NFAM1^-/-^ monocytes had decreased expression of multiple NFAT-target genes, including TNF-α (19), Il-12 (20), lymphotoxin-α (21), Cdk5r1 (22), Bcl2 (23) and Nr4a1 (24).

Although NFAT is best known for its role in regulating T cell activation, recent findings have suggested that NFAT also regulates activation of innate immune cells. For example, NFAT promotes bacterial and fungal activation of macrophages, dendritic cells and monocytes (3, 25, 26). Additionally, there are reports that NFAT is constitutively activate in macrophages, resulting in both enhanced TLR responses and inappropriate, disease-inducing cytokine production (18). Notably, we observed that NFAM1^-/-^ monocytes had decreased Nr4a1 expression in the absence of CD40L stimulation, suggesting that NFAM1 may promote constitutive NFAT activation. To identify the conditions under which NFAM1 triggers activation of NFAT, it will be important to determine if NFAM1 binds to a specific ligand, as ITAM containing receptors are typically activated by crosslinking. Experiments to identify potential ligands of NFAM1, as well as interrogation of downstream signaling will be the topic of future studies.

Lastly, we tested the impact of NFAM1 deletion on development of anti-CD40 induced colitis. Surprisingly, we did not observe protection against development of colon pathology as measured by weight loss, epithelial changes, mucosal gland loss and inflammation. Furthermore, despite dramatically decreased production of pro-inflammatory cytokines in CD40L-stimulated NFAM1^-/-^ monocytes, we observed only a minor decrease in serum levels of TNF-α and no decrease in serum levels of IL-1β, IL-6, or IL-12. There are multiple potential explanations for the discrepancy between our *in vitro* and *in vivo* results. For one, our *in vitro* experiments focused on the role of NFAM1 in monocytes, while there are multiple cell types that contribute to anti-CD40 colitis, not all of which may be impacted by NFAM1 deletion. Furthermore, the role of NFAM1 may be context dependent. In support of this hypothesis, thioglycolate-induced peritoneal macrophages from NFAM1-/- mice exhibited only a minor decrease in CD40L-induced TNF-α production, suggesting that the role of NFAM1 may be diminished in a pro-inflammatory environment. Further investigation into the role of NFAM1 in additional cell types and pro-inflammatory settings will provide further insight into the role of NFAM1 in disease.

In summary, we demonstrated that NFAM1 promotes pro-inflammatory responses in *in vitro*-stimulated monocytes and has a modest impact on TNF-α production *in vivo*. This novel role of NFAM1 warrants further investigation into the role of NFAM1 in additional autoimmune diseases.

## Data Availability Statement

The datasets presented in this study can be found in online repositories. The names of the repository/repositories and accession number(s) can be found in the article/**Supplementary Material**.

## Ethics Statement

The studies involving human participants were reviewed and approved by Sanford Burnham Prebys Medical Discovery Institute, La Jolla, CA and Boehringer Ingelheim Pharmaceuticals, Inc., 900 Ridgebury Road, Ridgefield, CT. The patients/participants provided their written informed consent to participate in this study. The animal study was reviewed and approved by the Institutional Animal Care and Use Committee at Sanford Burnham Prebys Medical Discovery Institute, La Jolla, CA, and Boehringer Ingelheim Pharmaceuticals, Inc., 900 Ridgebury Road, Ridgefield, CT, United States.

## Author Contributions

KJ designed experiments, carried out research, analyzed data and wrote the paper. AG designed experiments, carried out research, analyzed data and contributed to writing the paper. JL and SC designed experiments and contributed to writing the paper. MLM designed experiments. JPG, ES, NY, NJH, ACM, SEF, SSW, ERAG, and IY carried out research and analyzed data. DS designed experiments, carried out research, and analyzed data. JR designed NFAM1 DNA constructs. LL analyzed RNAseq data. JZ scored pathology samples. All authors contributed to the article and approved the submitted version.

## Funding

This work was funded by a research collaboration agreement with Boehringer Ingelheim Pharmaceuticals, Inc. and Sanford Burnham Prebys Medical Discovery Institute.

## Conflict of Interest

SC served as Principal Investigator of the contract. KWJ, JPG, ES, ACM, SEF, DS, SSW, ERAG, IY, JR, LL, JZ, MLM and JL were employees of Boehringer Ingelheim.

The authors disclose that financial support for this project was provided by Boehringer Ingelheim *via* a research contract to Sanford Burnham Prebys Medical Discovery Institute. Boehringer Ingelheim had the following involvement with the study: experimental design, data acquisition, analysis and writing the paper.

## Publisher’s Note

All claims expressed in this article are solely those of the authors and do not necessarily represent those of their affiliated organizations, or those of the publisher, the editors and the reviewers. Any product that may be evaluated in this article, or claim that may be made by its manufacturer, is not guaranteed or endorsed by the publisher.

## Supplementary Material

Supplementary Figure 1 NFAM1 is an NFAT activating Type I transmembrane receptor. **(A–C)** 293 cells were transfected with either untagged NFAM1 or NFAM1 bearing either an N-terminal or C-terminal FLAG-tag. Cell surface anti-FLAG staining is shown in **(A)**. Intracellular anti-FLAG staining is shown in **(B)**. Anti-NFAM1 western blot is shown in **(C)**. Data are representative of two independent experiments. Jurkat cells expressing an NFAT luciferase reporter were transfected with DNA constructs encoding the extracellular and transmembrane domain of CD8α fused to the cytoplasmic domain of either wildtype NFAM1 or NFAM1with an inactivated ITAM. Post transfection, cells were cultured in the absence or presence of anti-CD8α. Luciferase activity is shown in **(D)**. Data are representative of two independent experiments. Statistical significance is depicted as follows: **** indicates a P value of <0.0001.

Supplementary Figure 2 NFAM1^-/-^ monocytes produce reduced TNF-α in response to stimulation with CD40L, regardless of whether cells were primed with IFN-γ. Monocytes from 6 NFAM1^+/+^ mice and 6 NFAM1^-/-^ mice. were stimulated with CD40L with or without IFN-γ priming. Shown is quantification of TNF-α in the supernatant at 48 hours. Data are representative of two independent experiments. Statistical significance is depicted as follows: **** indicates a P value of <0.0001, * indicates a P value of <0.05 and ns indicates the comparison is not statistically significant.

Supplementary Figure 3 RNAseq analysis confirms that NFAM1^-/-^ monocytes have little to no defect in CD40L-induced expression of CD80, CD86, CD40, MHC-I and MHC-II. Monocytes from 4 NFAM1^+/+^ and 6 NFAM1^-/-^ mice were left unstimulated or stimulated with IFN-γ and CD40L. RNA was isolated and analyzed by RNAseq at 6 and 24 hours. Mean FPKM for select genes at 6 hours is shown in **(A–E)**. Statistical significance is depicted as follows: ** indicates a P value of <0.01 and ns indicates the comparison is not statistically significant.

Supplementary Figure 4 Thioglycolate-induced peritoneal macrophages from NFAM1^-/-^ mice produce reduced TNF-α in response to stimulation with IFN-γ and CD40L. 4 NFAM1^+/+^ and 4 NFAM1^-/-^ mice were given an intraperitoneal injection of 3% thioglycolate. On day 3, mice were sacrificed, peritoneal lavage was collected, and macrophages were isolated *via* centrifugation. Shown is quantification of TNF-α in the supernatant at 48 hours post stimulation with IFN-γ and CD40L. Data are combined from two independent experiments for a total of 8 NFAM1+/+ and 8 NFAM1-/- mice. Statistical significance is depicted as follows: * indicates a P value of <0.05 and ns indicates the comparison is not statistically significant.

Supplementary Figure 5 There is no significant difference in T cell-mediated cytokine production in NFAM1^-/-^ and NFAM1^+/+^ mice. **(A–C)** 10 NFAM1^-/-^ and 10 NFAM1^+/+^ mice were immunized at the base of the tail with OVA emulsified in complete Freund’s adjuvant. On day 10, inguinal lymph nodes were isolated and restimulated with 0, 11, 33.3 or 100 µg/mL of OVA. Mean IFN-γ production is shown in **(A)**, mean IL-17 production is shown in **(B)**, and mean TNF-α production is shown in **(C)**. Data are representative of two independent experiments. NS indicates the comparison is not statistically significant.

Supplementary Figure 6 NFAM1 promotes CD40L-induced cytokine and chemokine production in human monocytes. **(A–G)** Human monocytes from 1 donor were treated with Non-targeting (NT) CRISPR-RNP or NFAM1 CRISPR-RNP. Cells were plated in duplicate and stimulated with CD40L for 24 hours. Shown are NFAM1 protein expression **(A)** and MIP-1β **(B)**, TNF-α **(C)**, IL-8 **(D),** IL-1β **(E)**, IL-1Ra **(F)** and CCL22 **(G)** as measured in the supernatant by luminex.
