# Supplementary figures and images for: NFAM1 Promotes Pro-Inflammatory Cytokine Production in Mouse and Human Monocytes
Source: Front Immunol. 2022 Jan 13;12:773445. doi: 10.3389/fimmu.2021.773445 (PMC8793151; doi:10.3389/fimmu.2021.773445)

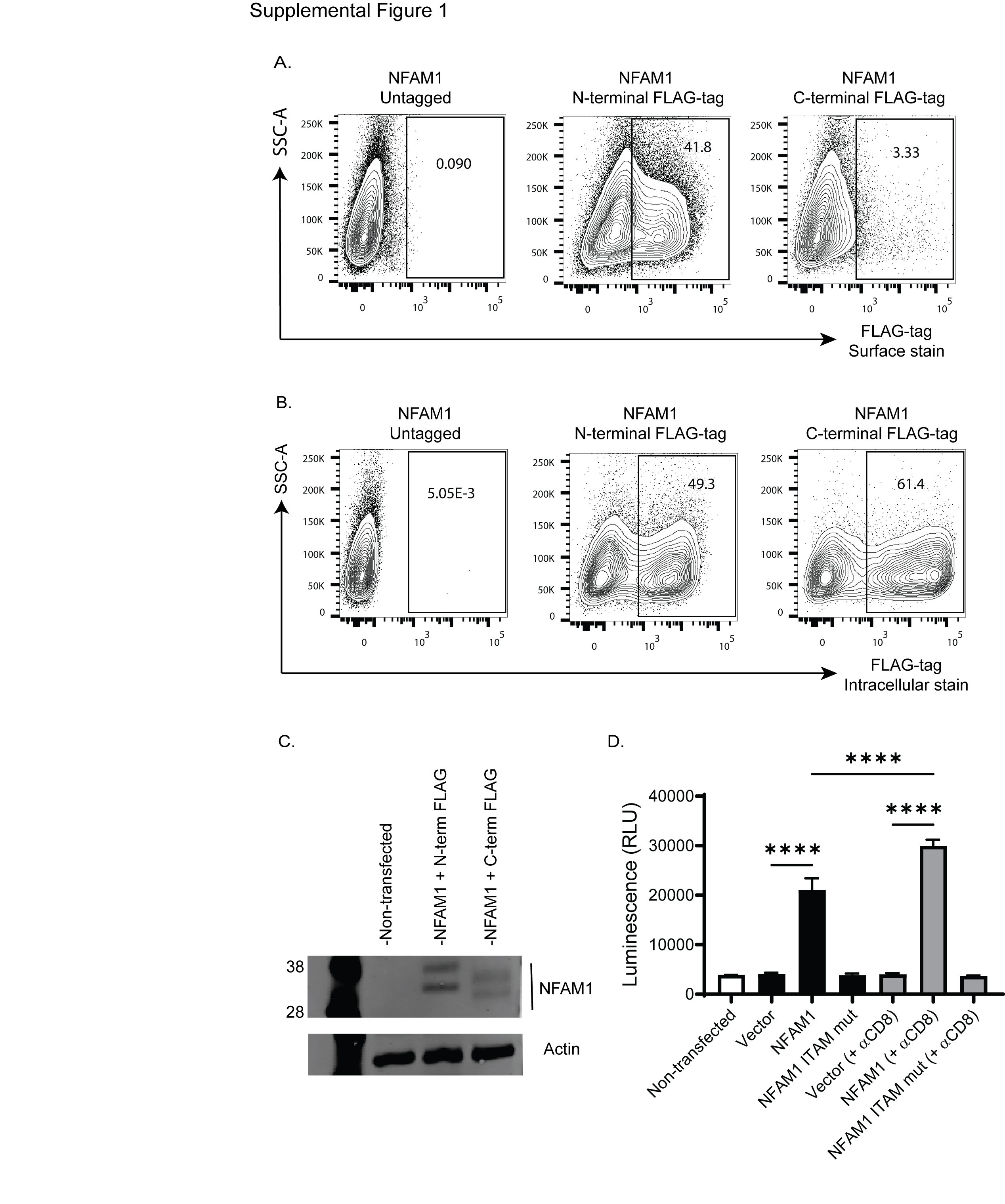

Supplement: Supplementary Figure 1 — NFAM1 is an NFAT activating Type I transmembrane receptor. (A–C) 293 cells were transfected with either untagged NFAM1 or NFAM1 bearing either an N-terminal or C-terminal FLAG-tag. Cell surface anti-FLAG staining is shown in (A). Intracellular anti-FLAG staining is shown in (B). Anti-NFAM1 western blot is shown in (C). Data are representative of two independent experiments. Jurkat cells expressing an NFAT luciferase reporter were transfected with DNA constructs encoding the extracellular and transmembrane domain of CD8α fused to the cytoplasmic domain of either wildtype NFAM1 or NFAM1with an inactivated ITAM. Post transfection, cells were cultured in the absence or presence of anti-CD8α. Luciferase activity is shown in (D). Data are representative of two independent experiments. Statistical significance is depicted as follows: **** indicates a P value of <0.0001. [file Image_1.jpeg]

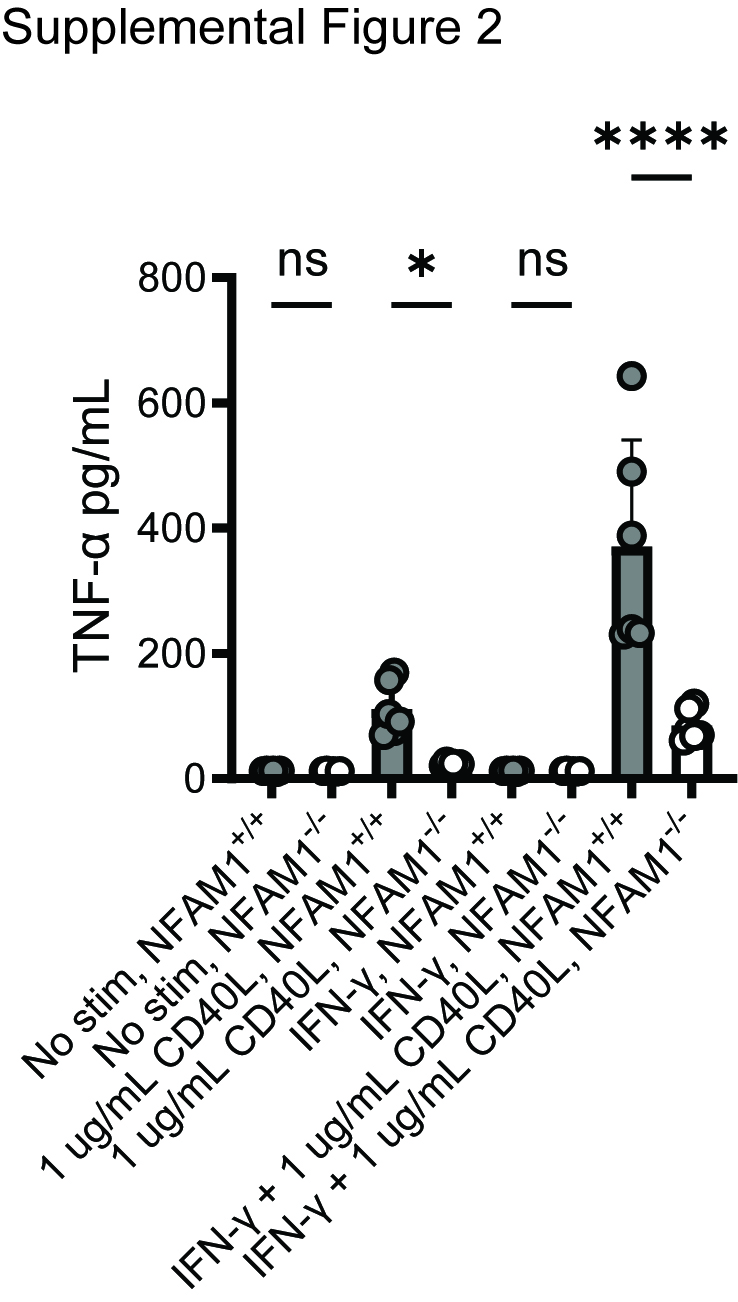

Supplement: Supplementary Figure 2 — NFAM1-/- monocytes produce reduced TNF-α in response to stimulation with CD40L, regardless of whether cells were primed with IFN-γ. Monocytes from 6 NFAM1+/+ mice and 6 NFAM1-/- mice. were stimulated with CD40L with or without IFN-γ priming. Shown is quantification of TNF-α in the supernatant at 48 hours. Data are representative of two independent experiments. Statistical significance is depicted as follows: **** indicates a P value of <0.0001, * indicates a P value of <0.05 and ns indicates the comparison is not statistically significant. [file Image_2.jpeg]

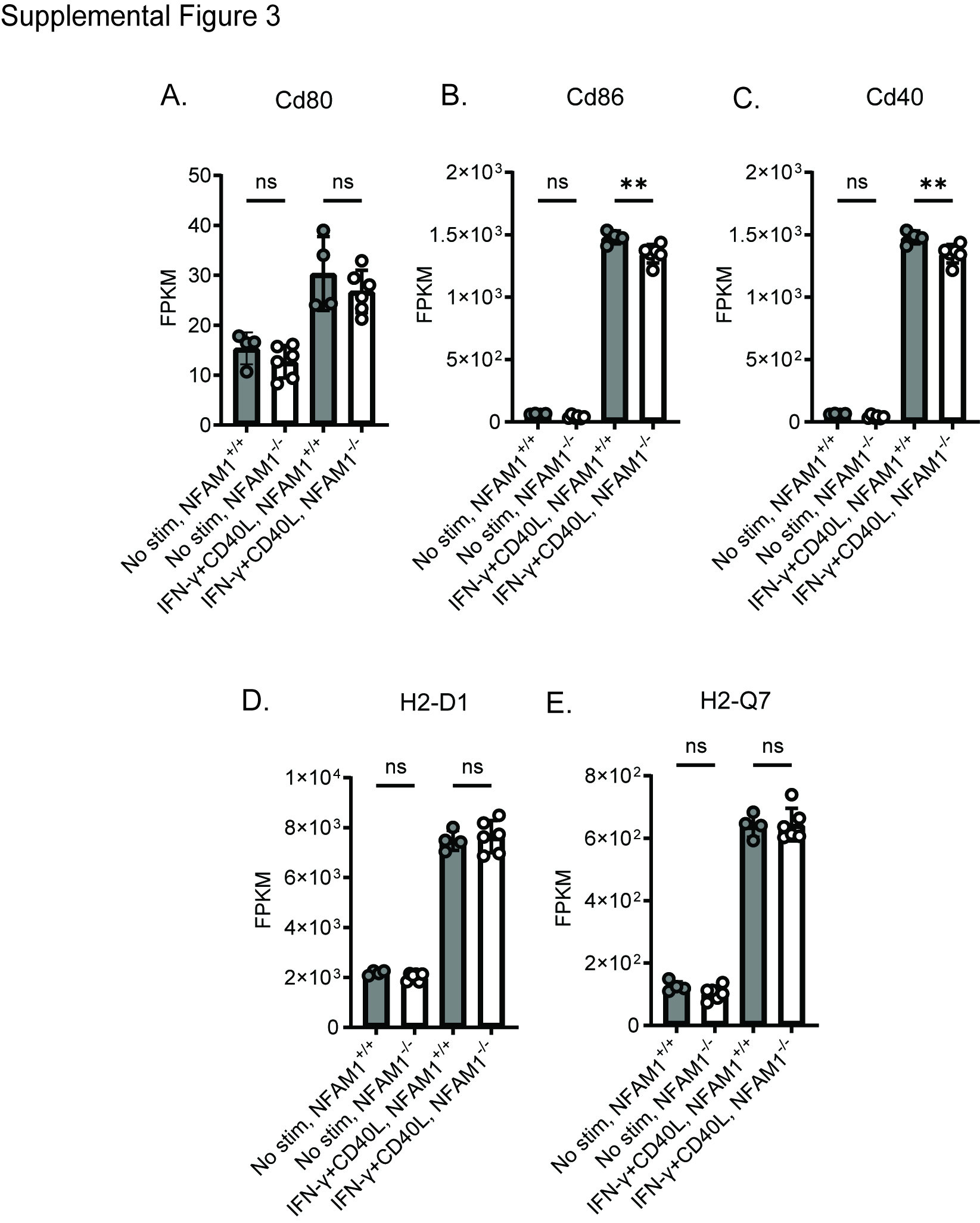

Supplement: Supplementary Figure 3 — RNAseq analysis confirms that NFAM1-/- monocytes have little to no defect in CD40L-induced expression of CD80, CD86, CD40, MHC-I and MHC-II. Monocytes from 4 NFAM1+/+ and 6 NFAM1-/- mice were left unstimulated or stimulated with IFN-γ and CD40L. RNA was isolated and analyzed by RNAseq at 6 and 24 hours. Mean FPKM for select genes at 6 hours is shown in (A–E). Statistical significance is depicted as follows: ** indicates a P value of <0.01 and ns indicates the comparison is not statistically significant. [file Image_3.jpeg]

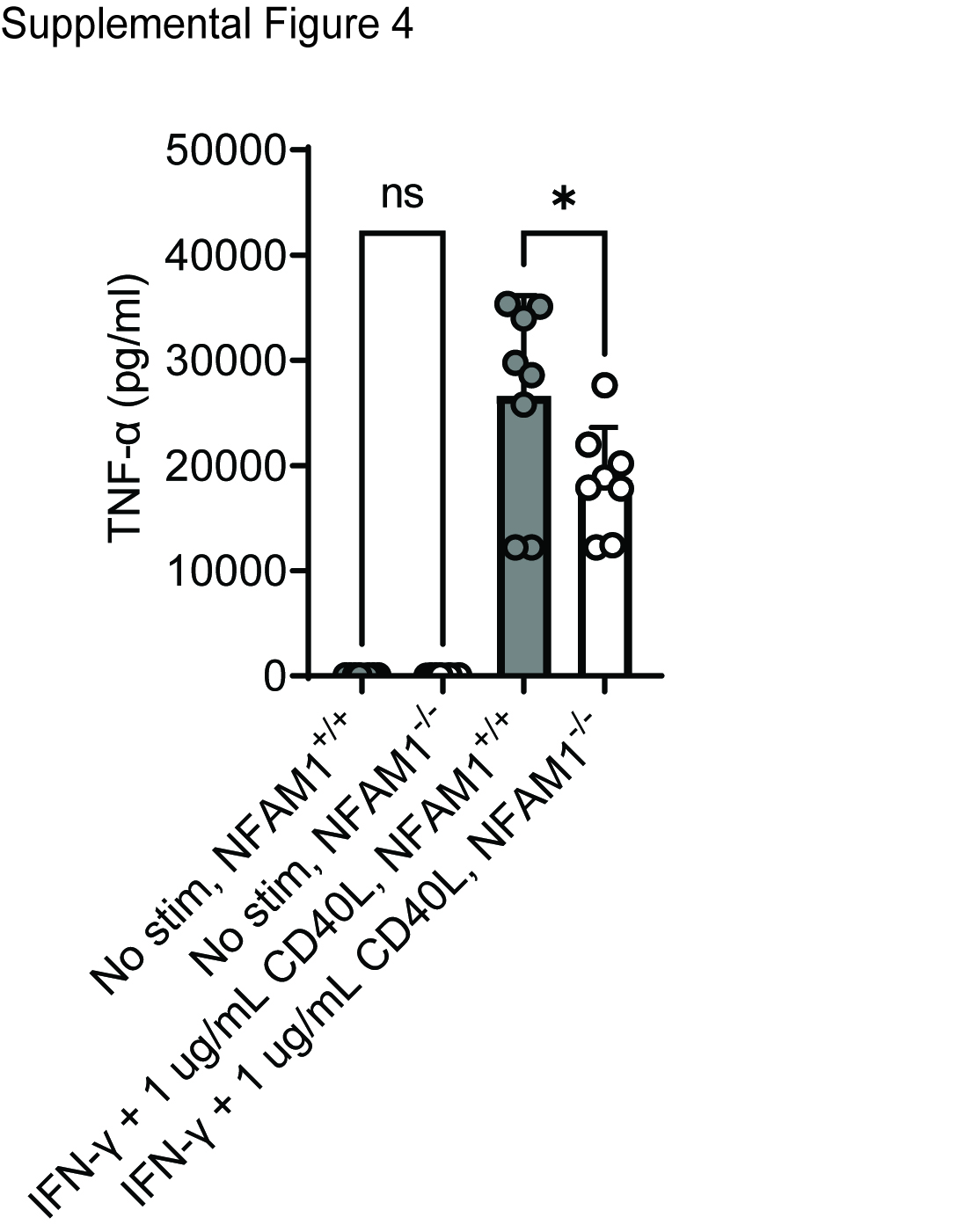

Supplement: Supplementary Figure 4 — Thioglycolate-induced peritoneal macrophages from NFAM1-/- mice produce reduced TNF-α in response to stimulation with IFN-γ and CD40L. 4 NFAM1+/+ and 4 NFAM1-/- mice were given an intraperitoneal injection of 3% thioglycolate. On day 3, mice were sacrificed, peritoneal lavage was collected, and macrophages were isolated via centrifugation. Shown is quantification of TNF-α in the supernatant at 48 hours post stimulation with IFN-γ and CD40L. Data are combined from two independent experiments for a total of 8 NFAM1+/+ and 8 NFAM1-/- mice. Statistical significance is depicted as follows: * indicates a P value of <0.05 and ns indicates the comparison is not statistically significant. [file Image_4.jpeg]

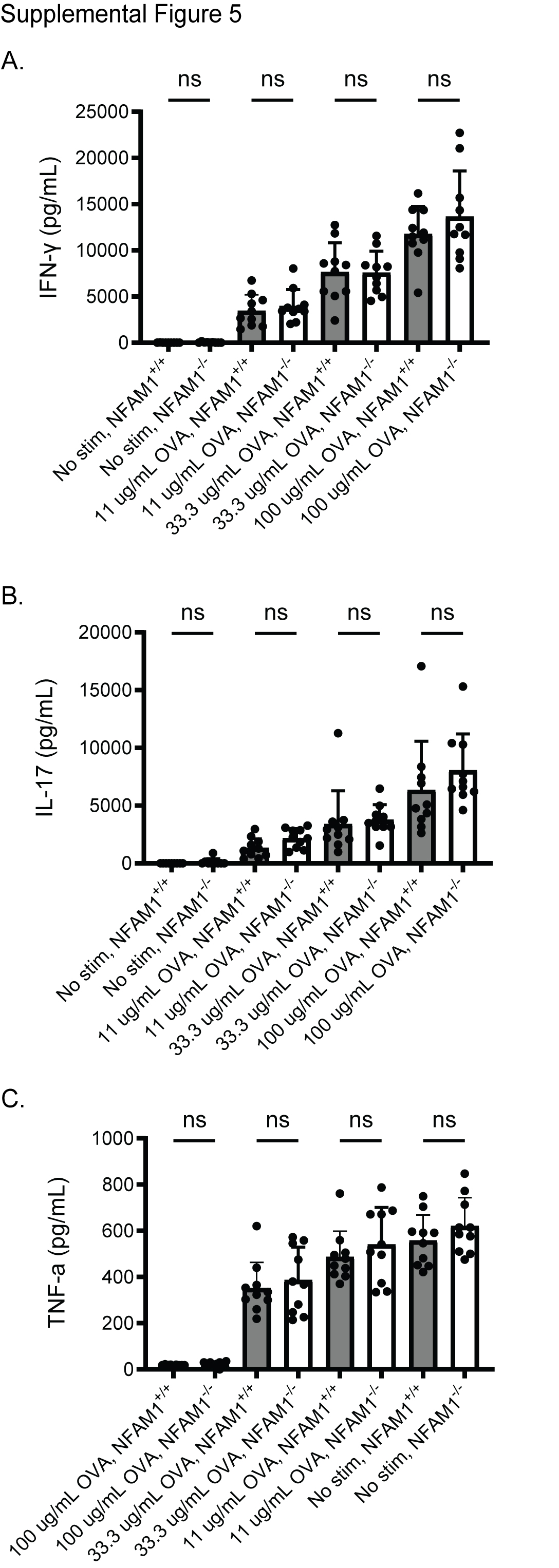

Supplement: Supplementary Figure 5 — There is no significant difference in T cell-mediated cytokine production in NFAM1-/- and NFAM1+/+ mice. (A–C) 10 NFAM1-/- and 10 NFAM1+/+ mice were immunized at the base of the tail with OVA emulsified in complete Freund’s adjuvant. On day 10, inguinal lymph nodes were isolated and restimulated with 0, 11, 33.3 or 100 µg/mL of OVA. Mean IFN-γ production is shown in (A), mean IL-17 production is shown in (B), and mean TNF-α production is shown in (C). Data are representative of two independent experiments. NS indicates the comparison is not statistically significant. [file Image_5.jpeg]

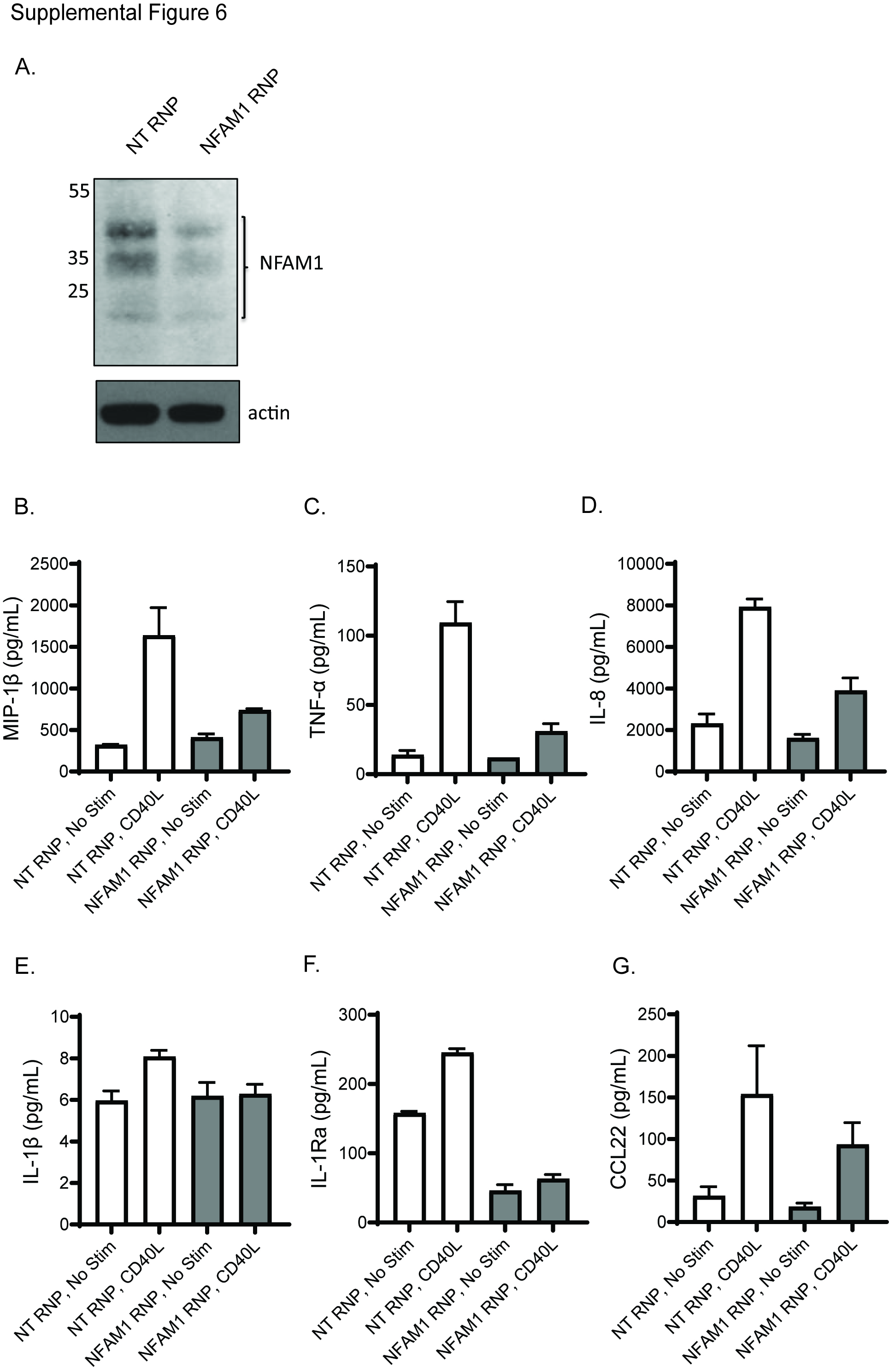

Supplement: Supplementary Figure 6 — NFAM1 promotes CD40L-induced cytokine and chemokine production in human monocytes. (A–G) Human monocytes from 1 donor were treated with Non-targeting (NT) CRISPR-RNP or NFAM1 CRISPR-RNP. Cells were plated in duplicate and stimulated with CD40L for 24 hours. Shown are NFAM1 protein expression (A) and MIP-1β (B), TNF-α (C), IL-8 (D), IL-1β (E), IL-1Ra (F) and CCL22 (G) as measured in the supernatant by luminex. [file Image_6.jpeg]
