# Supplementary material for: NFAM1 Promotes Pro-Inflammatory Cytokine Production in Mouse and Human Monocytes
Source: Front Immunol. 2022 Jan 13;12:773445. doi: 10.3389/fimmu.2021.773445 (PMC8793151; doi:10.3389/fimmu.2021.773445)
Supplement: Supplementary file 7 [file DataSheet_1.docx]

**Supplemental Methods**

NFAM1 inserts (sequence below) were cloned into pcDNA vector.

pcDNA_HuNFAM1_11387 (WT NFAM1. Used for Figure 1E)

**3’**ctagagccaccatggagaatcagcccgtgcgatggcgagcccttcccggccttccaaggcctcctggtctgcctgcagctccttggctgcttctgggcgtgctgttgctgcccggtaccctgaggctcgctggggggcagagcgtgactcacaccggcctgcccataatggcctcattggcaaacaccgccatctccttctcctgtaggatcacctatccctacactccccaattcaaggtgttcaccgtgtcctacttccacgaggatttgcagggccagaggtcccctaagaagcccaccaactgccaccctggcctcggcaccgagaaccagagccataccctggattgccaggtgaccctggtgctgcctggcgcttccgcaaccggtacatactactgctccgtgcactggccccattccaccgtgcgcggctccggcaccttcatcctggtgagggacgccggctatcgtgagccccctcagagtccacagaaactgctgctgttcggcttcactgggctcctctccgtacttagcgtcgtgggaacagccctcctgctgtggaacaagaagaggatgcgcggacccggaaaggatcccacccgcaaatgccctgacccccgatctgcctccagtcccaagcaacacccatccgaatctgtgtataccgcactgcaaaggcgggagaccgaggtgtacgcctgcattgagaacgaagacggctcttcccctaccgcaaagcagtcaccactgtcccaggagaggccacacaggttcgaagatgacggcgagcttaacctggtttatgagaacttgtaatgaa**5’**

pcDNA_HuNFAM1_11392 (Full length NFAM1 with C-terminal FLAG tag. Used for Supplemental Figure 1A-C).

**3’**ctagagccaccatggagaaccagcccgtaaggtggcgagccctgcctggcctccctcgaccccctgggttgccagcagccccatggctcctgctgggggtacttctgcttccaggaactctgcgcttggcaggcggtcaaagcgtgacacacaccggactgcccataatggcttctcttgccaataccgcaatctccttcagttgcaggattacctacccctacacaccccaattcaaggtgttcaccgtgtcctacttccacgaggacctccaggggcaaaggtcccccaaaaagcccaccaactgccatcccgggctcggcactgaaaaccaatcccacaccctggattgccaggtgaccctggtactgcccggagcatccgccaccggcacctactactgttccgtgcactggccccacagcaccgtcaggggctctggcacattcatcctggtgagggacgccggctacagggaaccccctcagtcaccccagaagctgctgcttttcggctttacaggactgctgtccgtgctctccgtggtgggaacagcccttcttctgtggaacaaaaagaggatgcgtggcccaggcaaggaccccacaaggaagtgccccgaccccaggtccgcctcctctcccaagcaacacccctccgagtccgtttacaccgccttgcaaaggcgtgagaccgaggtttacgcctgcatcgagaacgaagacggctccagccctactgctaagcaatcccccctgtcccaagaacgtccccataggtttgaggacgacggcgagctgaacctggtgtatgagaacttggctggctccgctgattacaaggacgatgacgacaaataatgaa**5’**

pcDNA_HuNFAM1_11391 (Full length NFAM1 with N-terminal FLAG tag. Used for Supplemental Figure 1A-C).

**3’**ctagagccaccatggagaaccagcccgtaaggtggcgagccctgcctggcctccctcgaccccctgggttgccagcagccccatggctcctgctgggggtacttctgcttccaggaactctgcgcttggcaggcggtcaatctgtaaccgactacaaagatgacgacgacaaggccggctccgcccaaagcgtgacacacaccggactgcccataatggcttctcttgccaataccgcaatctccttcagttgcaggattacctacccctacacaccccaattcaaggtgttcaccgtgtcctacttccacgaggacctccaggggcaaaggtcccccaaaaagcccaccaactgccatcccgggctcggcactgaaaaccaatcccacaccctggattgccaggtgaccctggtactgcccggagcatccgccaccggcacctactactgttccgtgcactggccccacagcaccgtcaggggctctggcacattcatcctggtgagggacgccggctacagggaaccccctcagtcaccccagaagctgctgcttttcggctttacaggactgctgtccgtgctctccgtggtgggaacagcccttcttctgtggaacaaaaagaggatgcgtggcccaggcaaggaccccacaaggaagtgccccgaccccaggtccgcctcctctcccaagcaacacccctccgagtccgtttacaccgccttgcaaaggcgtgagaccgaggtttacgcctgcatcgagaacgaagacggctccagccctactgctaagcaatcccccctgtcccaagaacgtccccataggtttgaggacgacggcgagctgaacctggtgtatgagaacttgtaatgaa**5’**

pcDNA_HuNFAM1_11434 (CD8 extracellular and transmembrane domains fused to WT NFAM intracellular domain. Used for Supplemental Figure 1D)

**3’**ctagagccaccatggccctgcccgtaaccgccctgctcctgcccctggcactgctgctgcatgccgccagacccagccagttcagggtgagtcctctggacaggacctggaaccttggcgagaccgtggagctcaagtgtcaggtgctcctcagtaaccccacgtccggatgcagctggctttttcagccccgaggtgcagcggccagcccgaccttcctgctgtacctgagccagaacaagcccaaagccgccgaggggcttgacacccagaggtttagtggcaagaggctgggcgacacattcgtgctgaccctgagcgacttcaggagggagaatgagggctactacttctgcagcgccttgtccaacagcatcatgtacttcagccacttcgtgcccgtgttcttgcccgccaaacctaccaccacaccggctccgagaccccctacgccagcgcccaccatcgctagccaacccttgtctctgaggcccgaagcgtgccgaccggcagccggaggcgccgtgcacaccagaggcttggacttcgcctgcgacatctacatctgggcaccgctcgctgggacgtgtggcgtgctcttgctgtcactcgtgataactctctggaacaagaaaaggatgcgcggaccaggcaaggatcccaccaggaagtgccccgatccgaggagcgccagcagccccaagcagcatccgagcgagagcgtctacacagccttgcagaggcgggagaccgaagtgtacgcctgcattgagaacgaggacggaagctcacctaccgcaaagcaatccccactgagtcaggaaagaccccataggttcgaagacgacggagagctgaacctggtttacgagaatctgtaatgaa**5’**

pcDNA_HuNFAM1_11435 (CD8 extracellular and transmembrane domains fused to NFAM1 intracellular domain with mutated ITAM. Used for Supplemental Figure 1D).

**3’**ctagagccaccatggccctgcccgtaaccgccctgctcctgcccctggcactgctgctgcatgccgccagacccagccagttcagggtgagtcctctggacaggacctggaaccttggcgagaccgtggagctcaagtgtcaggtgctcctcagtaaccccacgtccggatgcagctggctttttcagccccgaggtgcagcggccagcccgaccttcctgctgtacctgagccagaacaagcccaaagccgccgaggggcttgacacccagaggtttagtggcaagaggctgggcgacacattcgtgctgaccctgagcgacttcaggagggagaatgagggctactacttctgcagcgccttgtccaacagcatcatgtacttcagccacttcgtgcccgtgttcttgcccgccaaacctaccaccacaccggctccgagaccccctacgccagcgcccaccatcgctagccaacccttgtctctgaggcccgaagcgtgccgaccggcagccggaggcgccgtgcacaccagaggcttggacttcgcctgcgacatctacatctgggcaccgctcgctgggacgtgtggcgtgctcttgctgtcactcgtgataactctctggaacaagaaaaggatgcgcggaccaggcaaggatcccaccaggaagtgccccgatccgaggagcgccagcagccccaagcagcatccgagcgagagcgtcttcacagccttgcagaggcgggagaccgaagtgtttgcctgcattgagaacgaggacggaagctcacctaccgcaaagcaatccccactgagtcaggaaagaccccataggttcgaagacgacggagagctgaacctggtttacgagaatctgtaatgaa**5’**
